# Supplementary material for: Preparation of photonic molecular trains via soft-crystal polymerization of lanthanide complexes
Source: Nat Commun. 2022 Jul 5;13:3660. doi: 10.1038/s41467-022-31164-z (PMC9256636; doi:10.1038/s41467-022-31164-z)
Supplement: Supplementary file 1 — SUPPLEMENTARY INFO [file 41467_2022_31164_MOESM1_ESM.pdf]

Supporting information for

Preparation of photonic molecular trains in soft-crystal polymerization of lanthanide complexes

Pedro Paulo Ferreira da Rosa,[1] Yuichi Kitagawa,[2,3] Sunao Shoji,[2,3] Hironaga Oyama,[4] Keisuke Imaeda,[5] Naofumi Nakayama,[6] Koji Fushimi,[2] Hidehiro Uekusa,[4] Kosei Ueno,[5] Hitoshi Goto,[7] and Yasuchika Hasegawa[2,3]*

[1]Graduate School of Chemical Sciences and Engineering, Hokkaido University, Sapporo, Hokkaido 060-8628, Japan.

[2]Faculty of Engineering, Hokkaido University, Sapporo, Hokkaido 060-8628, Japan.

[3]Institute for Chemical Reaction Design and Discovery (WPI-ICReDD), Hokkaido University, Sapporo, Hokkaido 001-0021, Japan.

[4]School of Science, Tokyo Institute of Technology, Tokyo 152-8551, Japan.

[5]Faculty of Science, Hokkaido University, Sapporo, Hokkaido 060-0810, Japan.

[6]CONFLEX Corporation, Tokyo 108-0074, Japan.

[7]Information and Media Center, Toyohashi University of Technology, Toyohashi, Aichi 441-8580, Japan.

---

*To whom correspondence should be addressed.

E-mail: hasegaway@eng.hokudai.ac.jp

Supplementary Methods

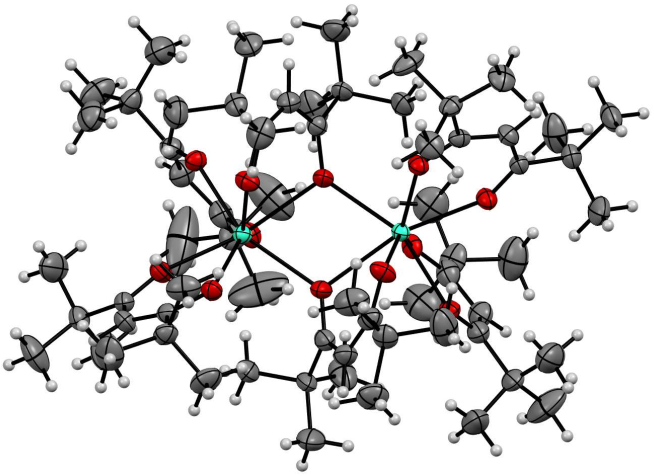

Supplementary Figure 1. Perspective view of $Ln_2(tmh)_6$ (CCDC 755629)[1], showing 50 % probability displacement ellipsoids. Gray spheres represent carbon; white spheres, hydrogen; red spheres, oxygen and green spheres, lanthanides.

Ln-dinuclear

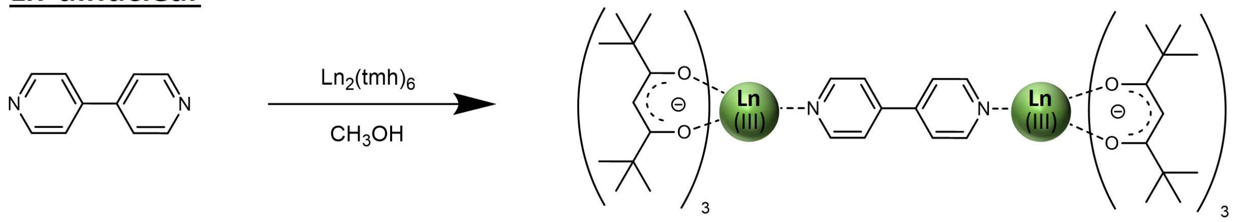

Ln-polymer

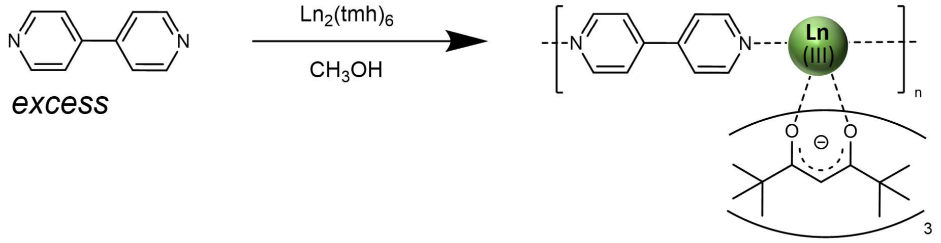

Supplementary Figure 2. Synthetic scheme of Ln-dinuclear (top) and Ln-polymer (bottom). Ln(III): Tb(III) or Dy(III), tmh: 2,2,6,6-tetramethyl-3,5-heptanedionato ($C_{11}H_{19}O_2$), 4,4'-bpy: 4,4'-bipyridine ($C_{10}H_8N_2$). All reactions were performed under reflux at 60 °C.

Supplementary Note 1: Crystal data

Similar dinuclear structures of Tb(III) and Dy(III) complexes were obtained showing three polymorphs (Supplementary Figure 3 and Supplementary Table 2). All measurements were made on a Rigaku XtaLAB PRO MM007 imaging plate area detector with graphite monochromatic Mo-K_{α} radiation. Corrections for decay and Lorentz-polarization effects were made using empirical absorption correction, solved by direct methods, and expanded using Fourier techniques. Non-hydrogen atoms were refined anisotropically using the SHELX system.[2] Hydrogen atoms were refined using the riding model. All calculations were performed using the crystal structure crystallographic and Olex 2 software package.[3] The CIF data was confirmed by the checkCIF/PLATON service. CCDC-2104176 (for $[Tb_2(tmh)_6(4,4'\text{-}bpy)]$), CCDC-2104175 (for $[Dy_2(tmh)_6(4,4'\text{-}bpy)]$), CCDC-2104174 (for $[Tb(tmh)_3(4,4'\text{-}bpy)]_n$), contain the supplementary crystallographic data for this paper. These data can be obtained free of charge from The Cambridge Crystallographic Data Centre via www.ccdc.cam.ac.uk/data_request/cif.

The powder diffraction data for transformed soft-crystals were recorded at room temperature on a Rigaku SmartLab diffractometer with Cu-K_{α} radiation and a D/teX Ultra detector covering 5–45° (2ϑ). The diffraction data were collected at room temperature. The powder simulation patterns for $[Ln_2(tmh)_6(4,4'\text{-}bpy)]$ (Ln: Tb(III) and Dy(III)) and $[Ln(tmh)_3(4,4'\text{-}bpy)]_n$ were generated using Mercury 2021.1 from the previously described single crystal X-ray structures collected at -150 °C.

Supplementary Table 1. Crystallographic data

	$[Dy_2(tmh)_6(4,4'\text{-bpy})]$	$[Tb_2(tmh)_6(4,4'\text{-bpy})]$	$[Tb(tmh)_3(4,4'\text{-bpy})]_n$
Chemical formula	$C_{76}H_{122}N_2O_{12}Dy_2$	$C_{76}H_{122}N_2O_{12}Tb_2$	$C_{43}H_{65}N_2O_6Tb$
F000	1640.0	3272.0	864.89
Crystal system	Triclinic	Triclinic	Monoclinic
Space group	*P -1* (no. 2)	*P -1* (no. 2)	$P2_1/c$ (no. 14)
a / Å	9.74380(10)	11.9730(2)	11.8300(3)
b / Å	11.9357(2)	19.4913(3)	34.6307(7)
c / Å	37.0588(7)	36.9619(5)	12.0379(3)
α / deg	96.3150(10)	93.4770(10)	90
β / deg	93.6220(10)	96.3170(10)	118.160(3)
γ / deg	101.3280(10)	101.407(2)	90
Volume / $Å^3$	4184.37(12)	8373.9(2)	4347.9(2)
Z	2	2	4
T / °C	-150	-150	-150
R_1[a] / %	4.99	8.57	3.31
wR_2[b] / %	13.92	18.04	9.98

[a] $R_1 = \Sigma||F_0| - |F_c|| / \Sigma|F_0|$.

[b] $wR_2 = [\Sigma w(F_0^2 - F_c^2)^2 / \Sigma w(F_0^2)^2]^{1/2}$.

Supplementary Note 2: Polymorphs

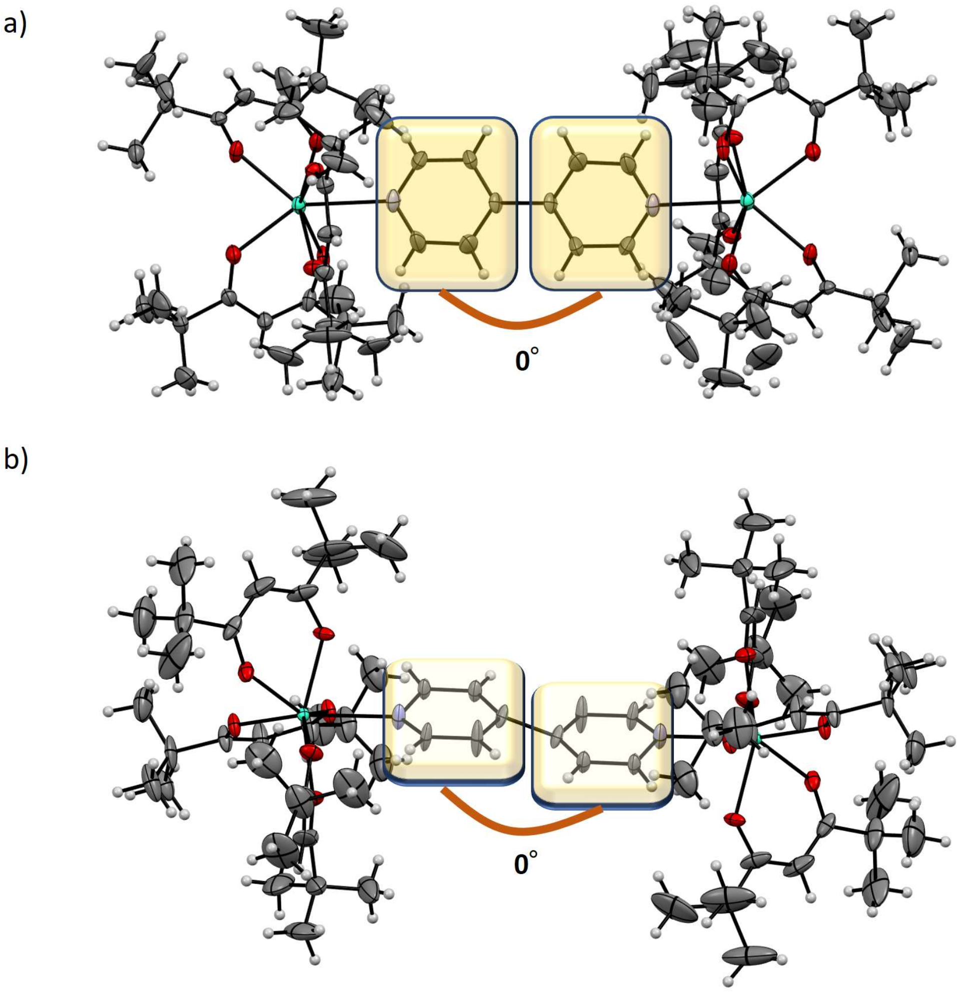

Supplementary Figure 3. Perspective views of $[Ln_2(tmh)_6(4,4'\text{-bpy})]$ (Ln: Tb(III) or Dy(III)) dinuclear complex of a) polymorph A and b) polymorph B showing 50% probability displacement ellipsoids and angle between pyridine planes. Gray spheres represent carbon; white spheres, hydrogen; red spheres, oxygen; blue spheres, nitrogen and green spheres, terbium or dysprosium. The planes of the bpy ligands are shown in yellow. tmh, 2,2,6,6-tetramethyl-3,5-heptanedionato; 4,4'-bpy, 4,4'-bipyridine.

CCDC-2106282 (for $[Tb_2(tmh)_6(4,4'\text{-bpy})]$-A), CCDC-2106281 (for $[Dy_2(tmh)_6(4,4'\text{-bpy})]$-A), CCDC-2106279 (for $[Tb_2(tmh)_6(4,4'\text{-bpy})]$-B), CCDC-2106280 (for $[Dy_2(tmh)_6(4,4'\text{-bpy})]$-B), contain the supplementary crystallographic data for these dinuclear polymorphs. These data can be obtained free of charge from The Cambridge Crystallographic Data Centre via www.ccdc.cam.ac.uk/data_request/cif.

Supplementary Table 2. Crystallographic data for dinuclear polymorphs

	[Dy_2(tmh)$_6$(4,4'-bpy)] - **A**	[Tb_2(tmh)$_6$(4,4'-bpy)] - **A**	[Dy_2(tmh)$_6$(4,4'-bpy)] - **B**	[Tb_2(tmh)$_6$(4,4'-bpy)] - **B**
Chemical formula	$C_{76}H_{122}N_2O_{12}Dy_2$	$C_{76}H_{122}N_2O_{12}Tb_2$	$C_{76}H_{122}N_2O_{12}Dy_2$	$C_{76}H_{122}N_2O_{12}Tb_2$
F000	1640.0	1636.0	820.0	818.0
Crystal system	Monoclinic	Monoclinic	Triclinic	Triclinic
Space group	$P2_1/n$ (no. 14)	$P2_1/n$ (no. 14)	*P -1* (no. 2)	*P -1* (no. 2)
a / Å	9.9349(2)	9.9131(4)	9.7319(2)	9.7401(2)
b / Å	21.1409(5)	21.1864(7)	11.9099(3)	11.9471(2)
c / Å	19.7546(4)	19.8214(7)	18.6753(5)	18.6244(5)
α / deg	90	90	89.770(2)	89.515(2)
β / deg	101.748(2)	101.838(4)	80.651(2)	80.412(2)
γ / deg	90	90	79.025(2)	78.8630(10)
Volume / $Å^3$	4062.20(15)	4074.4(3)	2095.95(9)	2096.10(8)
Z	4	4	2	2
T / °C	-150	-150	-150	-150
R_1[a] / %	2.47	8.14	4.31	6.85
wR_2[b] / %	5.82	16.32	11.05	18.88

[a] $R_1 = \Sigma||F_0| - |F_c|| / \Sigma|F_0|$.

[b] $wR_2 = [\Sigma w(F_0^2 - F_c^2)^2 / \Sigma w(F_0^2)^2]^{1/2}$.

Supplementary Note 3: SHAPE calculations

In order to obtain the closest coordination geometry around the Ln(III) centres, continuous shape measurements (CShM) calculations were performed using SHAPE.[4] The S_{CShM} criterion represents the degree of deviation from ideal coordination structure, and is given by Supplementary Equation (1):

$$S_{\mathrm{CShM}} = \min \frac{\sum_k^N |Q_k - P_k|^2}{\sum_k^N |Q_k - Q_0|^2} \times 100 \quad (1)$$

where Q_k is the vertices of an actual structure, Q_0 is the center of mass of an actual structure, P_k is the vertices of an ideal structure, and N is the number of vertices. The estimated S_{CShM} values of the Ln(III) complexes are summarized in Supplementary Table 3 and Supplementary Table 4. Based on the minimum value of S_{CShM}, the pseudo-coordination polyhedral structures of all seven-coordinated dinuclear Ln(III) complexes were categorized to be 7-MCO (monocapped-octahedron, point group: C_{3v}). For the eight-coordinated Tb(III) coordination polymer, the structure was categorized to be 8-SAP (Square antiprism, point group: D_{4d}). The coordination geometry of the seven-coordinated dinuclear complexes is more asymmetric than that of eight-coordinated Tb(III) coordination polymer.

Supplementary Table 3. Calculated S_{CShM} values for seven-coordinated Ln(III) complexes

Complex	$S_{7\text{-MCO}}$	$S_{7\text{-MCTP}}$	$S_{7\text{-PBP}}$
Tb-dinuclear1	0.63128	1.31333	11.17713
Tb-dinuclear2	0.83202	0.94406	9.66405
Dy-dinuclear1	0.49079	1.53628	11.18781
Dy-dinuclear2	0.79385	0.81259	10.28508
Tb-dinuclearA	0.96587	1.02542	9.82093
Dy-dinuclearA	0.91235	1.01750	9.84488
Tb-dinuclearB	0.57754	1.25175	10.91711
Dy-dinuclearB	0.53983	1.32892	10.92708

$S_{7\text{-MCO}}$, $S_{7\text{-MCTP}}$, $S_{7\text{-PBP}}$ are S values calculated for monocapped-octahedron (Point group: C_{3v}), monocapped-trigonal prism (Point group: C_{2v}) and pentagonal-bipyramid (Point group: D_{5h}), respectively.

Supplementary Table 4. Calculated S_{CShM} values for Tb(III) coordination polymer

Complex	$S_{8\text{-SAP}}$	$S_{8\text{-TDD}}$	$S_{8\text{-CU}}$
Tb-polymer	0.68469	2.84109	10.47320

$S_{8\text{-SAP}}$, $S_{8\text{-TDD}}$, $S_{8\text{-CU}}$ are S values calculated for square antiprism (Point group: D_{4d}), triangular dodecahedron (Point group: D_{2d}) and cube (Point group: O_h), respectively.

Supplementary Note 4: Structural transformation

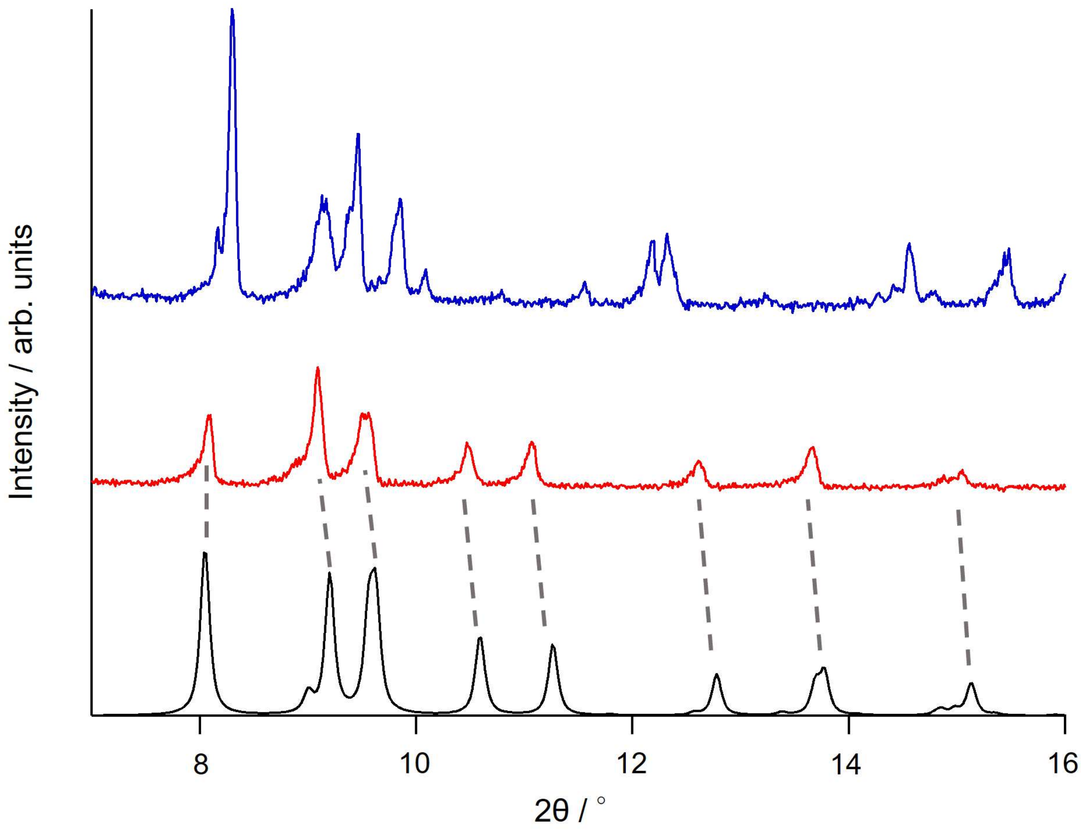

Supplementary Figure 4. Powder X-ray diffraction patterns of Tb-dinuclear (I: initial state, blue line), Tb-dinuclear under pyridine (py) vaporisation (II: red line) and the simulated pattern of [Tb(tmh)$_3$(py)$_2$] (CCDC-1979574)[5] (black line). Dashed lines included for easier identification of matched structures. tmh, 2,2,6,6-tetramethyl-3,5-heptanedionato; py, pyridine.

The simulated pattern of [Tb(tmh)$_3$(py)$_2$] was obtained from its single-crystal X-ray analyses at -150°C, while the PXRD measurements were performed at room temperature. This is the reason for the observed peak shifts derived from lattice expansions.

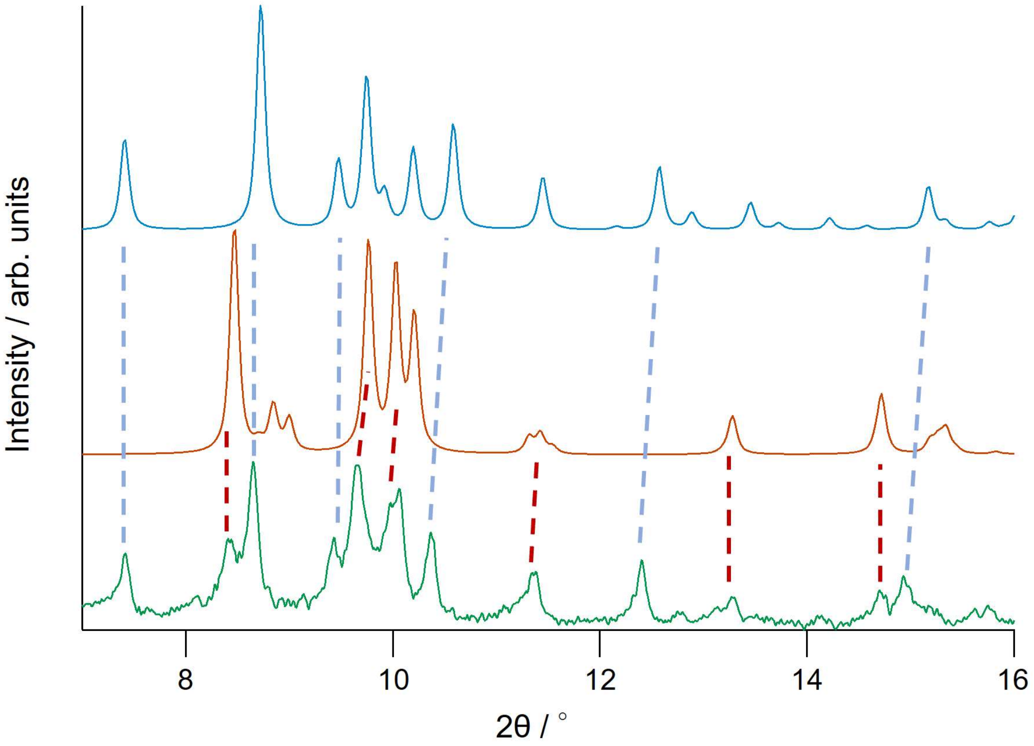

Supplementary Figure 5. Powder X-ray diffraction patterns of Tb-dinuclear after py removal (III: green line), the simulated pattern of $[Tb(tmh)_3(py)_1]$ (CCDC-1979573)[6] (sky blue line) and the simulated pattern of Tb-polymer (dark red line). Dashed lines included for easier identification of matched structures. tmh, 2,2,6,6-tetramethyl-3,5-heptanedionato; py, pyridine.

The simulated pattern of $[Tb(tmh)_3(py)_1]$ and Tb-polymer were obtained from their single-crystal X-ray analyses at -150°C, while the PXRD measurements were performed at room temperature. This is the reason for the observed peak shifts derived from lattice expansions. The final state is composed of a mix of Tb-polymer and $[Tb(tmh)_3(py)_1]$, as a byproduct.

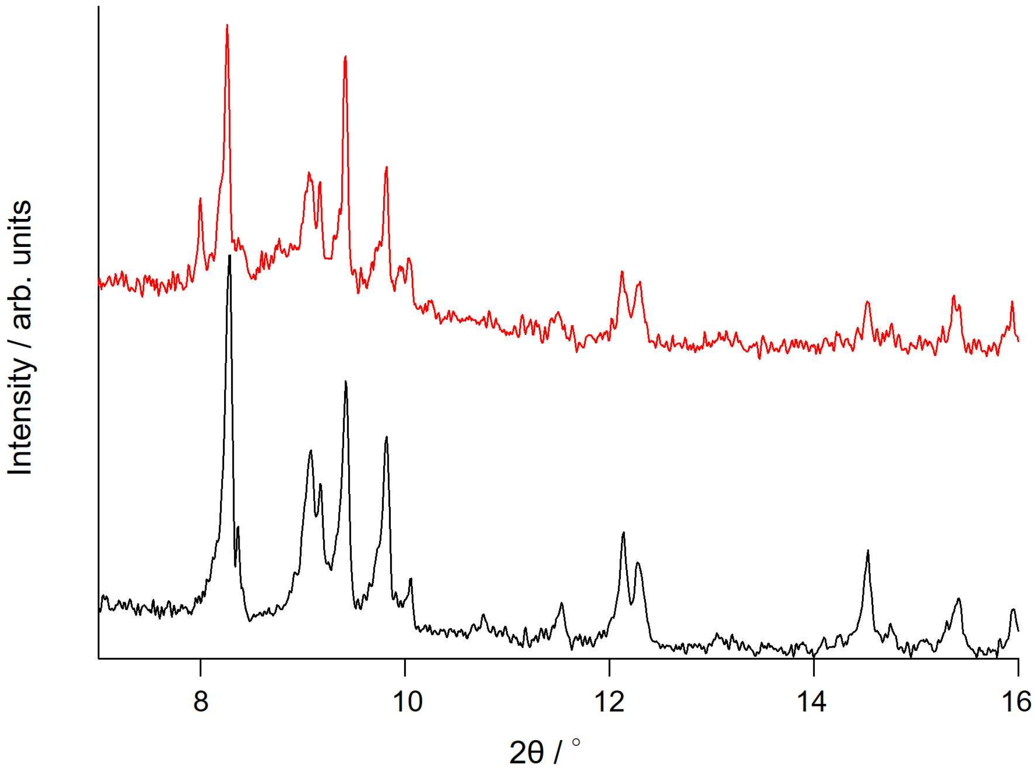

Supplementary Figure 6. Powder X-ray diffraction patterns of Tb-dinuclear after transformation under py (black line) and Tb-dinuclear after attempted transformation under methanol (red line).

Same patterns were observed, indicating no structural transformation.

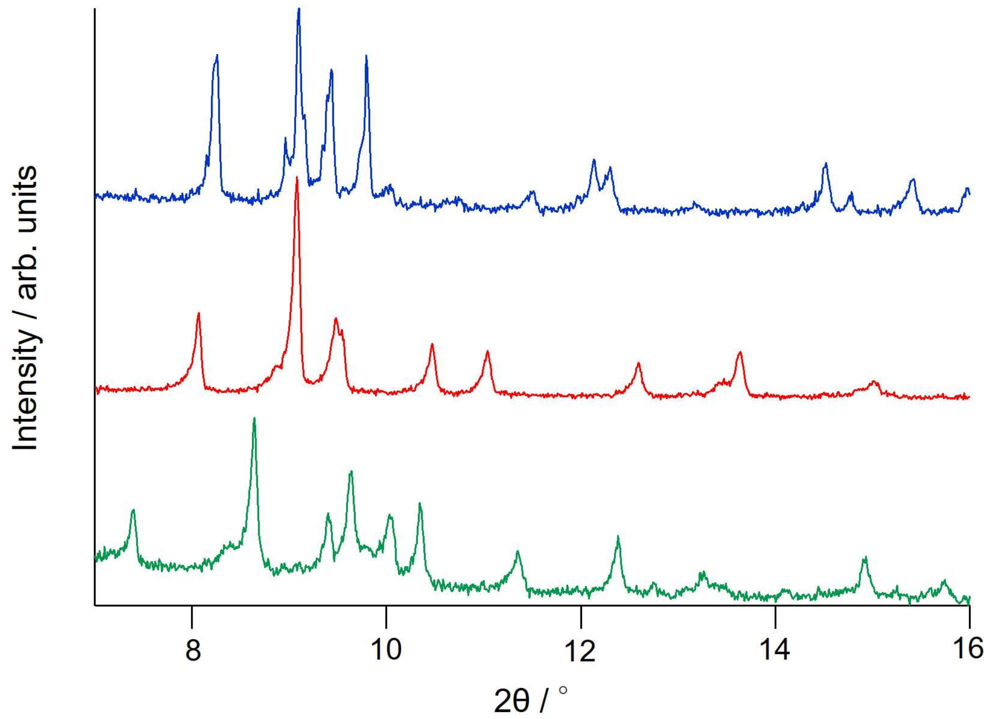

Supplementary Figure 7. Powder X-ray diffraction patterns of Dy-dinuclear before py vaporisation (I: initial state, blue line), under py vaporisation (II: red line) and after py removal (III: green line).

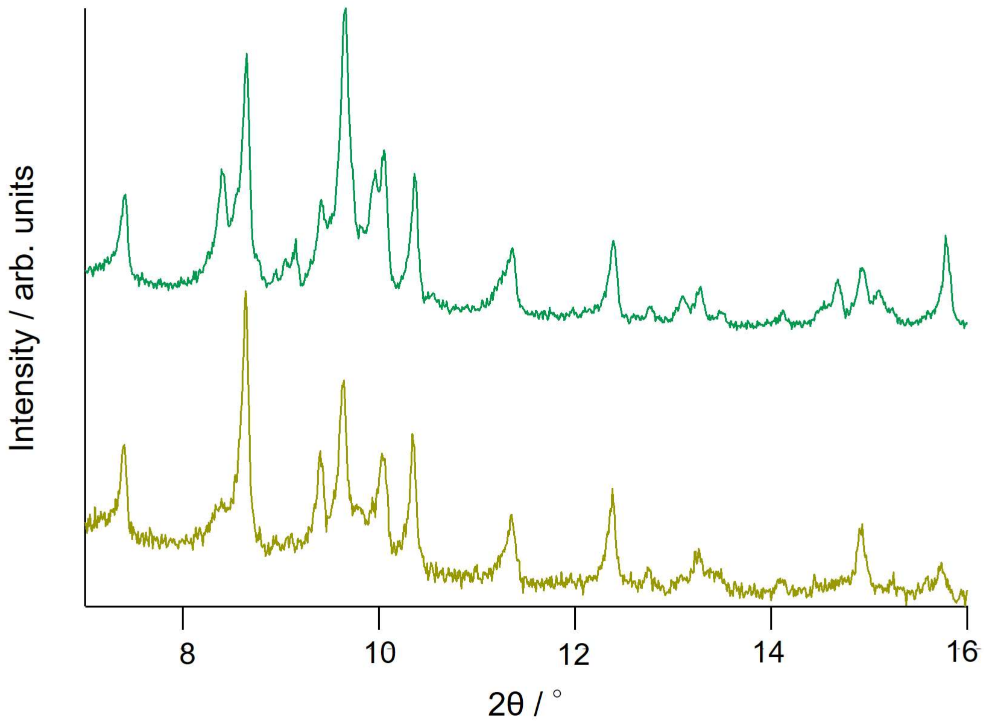

Supplementary Figure 8. Powder X-ray diffraction patterns of transformed Dy-dinuclear (dark yellow line) and transformed Tb-dinuclear (green line).

Similar structures obtained for the transformed Tb-dinuclear and Dy-dinuclear complexes via py vaporization. This indicates the successful polymerization of Dy-dinuclear.

Supplementary Note 5: Intermediate formation in py vaporisation

To investigate the formation of the intermediate and its importance to the structural polymerization, we performed density functional theory calculations (B3LYP-D3, basis set: SDD (MWB28) for Tb(III) and 6-31G(d) for other elements) in vacuum and pyridine (py) solvent (PCM) based on the Tb-dinuclear structure with one inserted py molecule in four different positions (Supplementary Figure 9). Supplementary Table 5 shows the coordination bond lengths for 4,4'-bpy and py with Tb(III), and the total energy of the system (normalized at type 1). It can be concluded that the type 1 is the most stable configuration for the structure with insertion of one py molecule.

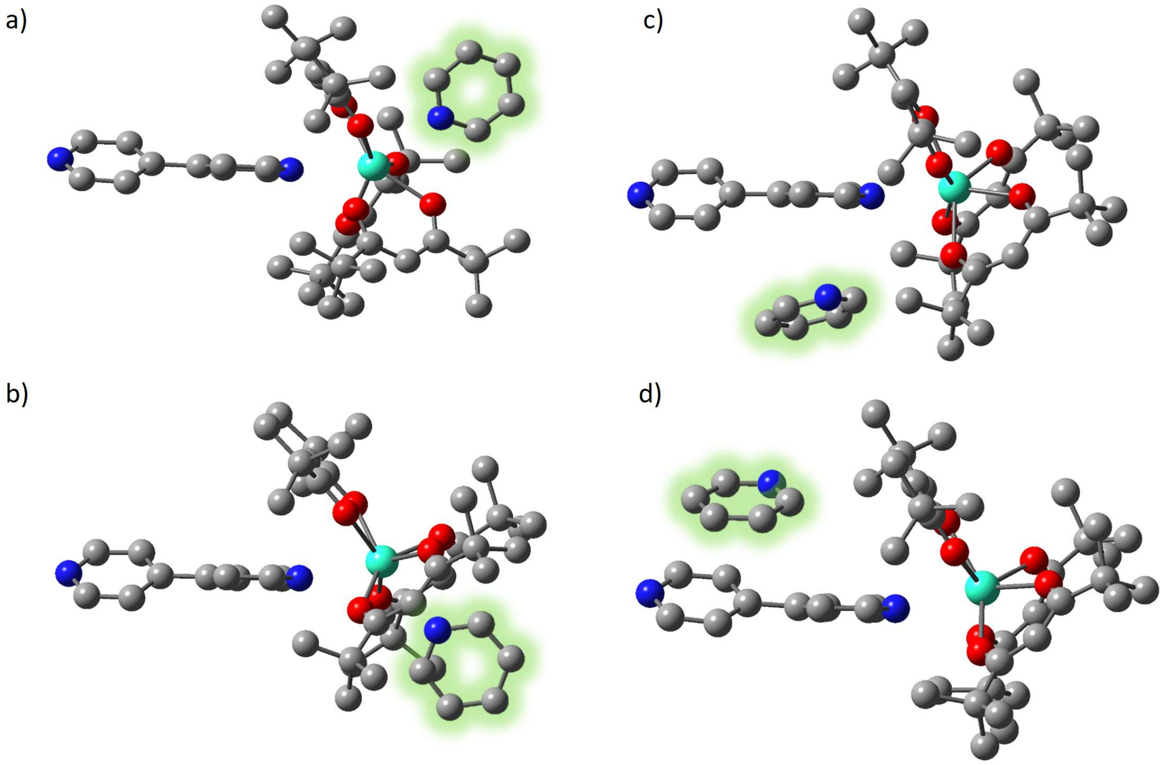

Supplementary Figure 9. Perspective views of structure based on Tb-dinuclear with one py molecule inserted on a) type 1: the rear of 4,4'-bpy from the top; b) type 2: the rear of 4,4'-bpy from the bottom; c) type 3: the front of 4,4'-bpy from the bottom; d) type 4: the front of 4,4'-bpy from the top. One $Tb(tmh)_3$ centre of the dinuclear structure and hydrogen atoms were omitted for clarity. Gray spheres represent carbon; red spheres, oxygen; blue spheres, nitrogen and green spheres, terbium. Py molecules are highlighted in green. tmh, 2,2,6,6-tetramethyl-3,5-heptanedionato; 4,4'-bpy, 4,4'-bipyridine; py, pyridine.

Supplementary Table 5. Coordination bond lengths and total system energy for each configuration with one inserted py

	vacuum			Pyridine (PCM)		
	Tb-N_{bpy} / Å	Tb-N_{py} / Å	Δ*E* / kcal.mol^{-1}	Tb-N_{bpy} / Å	Tb-N_{py} / Å	Δ*E* / kcal.mol^{-1}
Type 1	2.589	2.629	0	2.589	2.636	0
Type 2	2.614	2.730	1.46	2.615	2.714	0.73
Type 3	2.583	5.038	7.50	2.588	5.186	6.25
Type 4	2.592	6.739	6.77	2.591	6.805	5.20

Next, the coordination exchange between the second py and 4,4'-bpy takes place. Based on the most stable structure obtained with one inserted py (type 1), we performed new structure optimizations with the insertion of a second py at 3 different positions (Supplementary Figure 10). Supplementary Table 6 shows the coordination bond lengths for 4,4'-bpy and py with Tb(III), and the total energy of the system (normalized at type 1+3). It can be concluded that the type 1+3 is the most stable configuration for the structure with insertion of two py molecules.

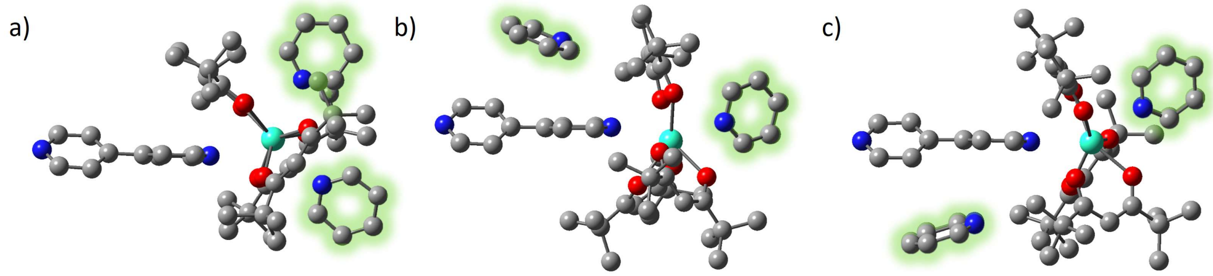

Supplementary Figure 10. Perspective views of structure based on Tb-dinuclear with one inserted py (type 1) with a new py inserted on a) type 1+2: the rear of 4,4'-bpy from the bottom; b) type 1+3: the front of 4,4'-bpy from the top; c) type 1+4: the front of 4,4'-bpy from the bottom. One $Tb(tmh)_3$ centre of the dinuclear structure and hydrogen atoms were omitted for clarity. Gray spheres represent carbon; red spheres, oxygen; blue spheres, nitrogen and green spheres, terbium. Py molecules are highlighted in green. tmh, 2,2,6,6-tetramethyl-3,5-heptanedionato; 4,4'-bpy, 4,4'-bipyridine; py, pyridine.

Supplementary Table 6. Coordination bond lengths and total system energy for each configuration with two inserted pys

	vacuum				Pyridine (PCM)			
	Tb-N_{bpy} / Å	Tb-N_{py1}/ Å	Tb-N_{py2}/ Å	Δ*E* / kcal.mol^{-1}	Tb-N_{bpy} / Å	Tb-N_{py} / Å	Tb-N_{py2}/ Å	Δ*E* / kcal.mol^{-1}
Type 1+2	2.739	2.856	2.786	0.91	2.775	2.842	2.812	1.00
Type 1+3	2.608	6.253	2.606	0	2.603	6.284	2.615	0
Type 1+4	2.589	6.088	2.630	2.20	2.594	6.054	2.636	1.29

The second py approaches to molecule from the same side of 4,4'-bpy. (Supplementary Figure 10 and Supplementary Table 6). This suggests the ligand coordination exchange from 4,4'-bpy to the newly inserted py. In summary, after the second py coordinates to the Tb(tmh)$_3$ centre, 4,4'-bpy is decoordinated, although present in the crystal structure (Supplementary Figure 11 and Supplementary Table 6).

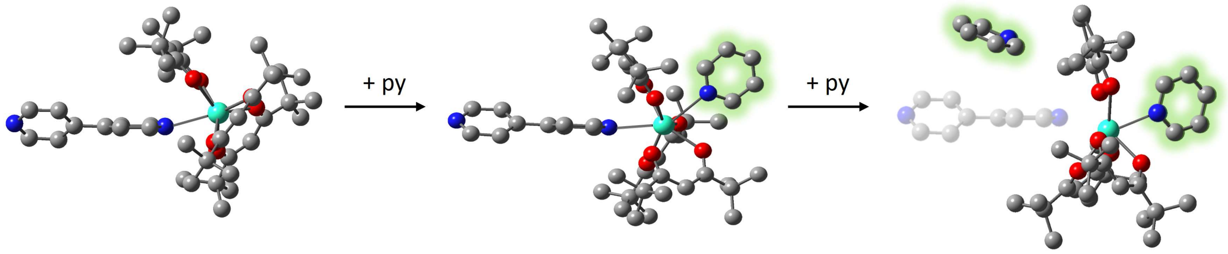

Supplementary Figure 11. Proposed structural transformation with the addition of py. One Tb(tmh)$_3$ centre of the dinuclear structure and hydrogen atoms were omitted for clarity. Gray spheres represent carbon; red spheres, oxygen; blue spheres, nitrogen and green spheres, terbium. Py molecules are highlighted in green. tmh, 2,2,6,6-tetramethyl-3,5-heptanedionato; py, pyridine.

Supplementary Note 6: Reversibility of polymerization

To investigate the reversibility of transformation from Ln-polymer (coordination polymer) to Ln-dinuclear (dinuclear complex), we performed calorimetric analyses using TG-DTA and DSC measurements of Tb-dinuclear and Tb-polymer (Supplementary Figure 12).

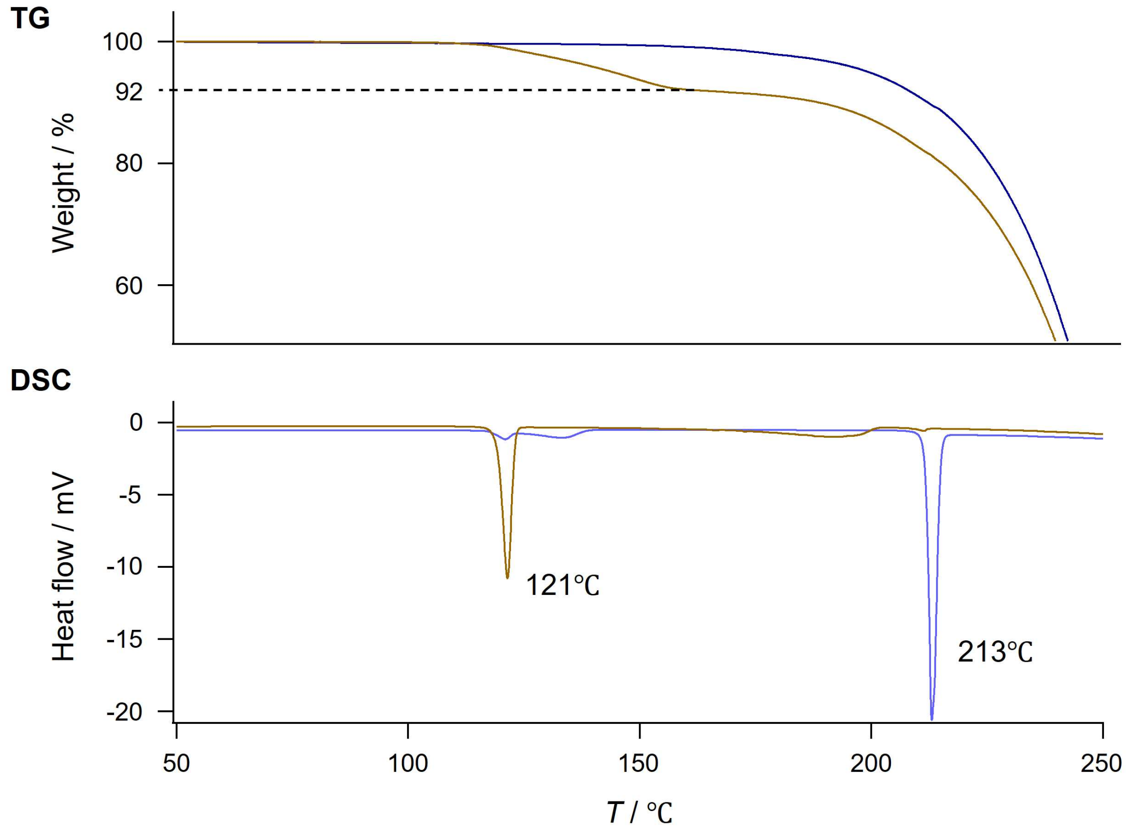

Supplementary Figure 12. Thermogravimetry (TG: top) and Differential Scanning Analysis (DSC: bottom) of Tb-dinuclear (blue line) and Tb-polymer (brown line).

An endothermic peak at 120 °C with correspondent 8% weight loss were observed for the Tb-polymer. This thermodynamic phenomenon is caused by elimination of coordinated organic molecules. The endothermic peak at 210 °C for the Tb-dinuclear is correspondent to the decomposition of the molecule. Based on thermodynamic analyses, we performed PXRD analyses of the Tb-polymer under heating at 130 °C for 20 min.

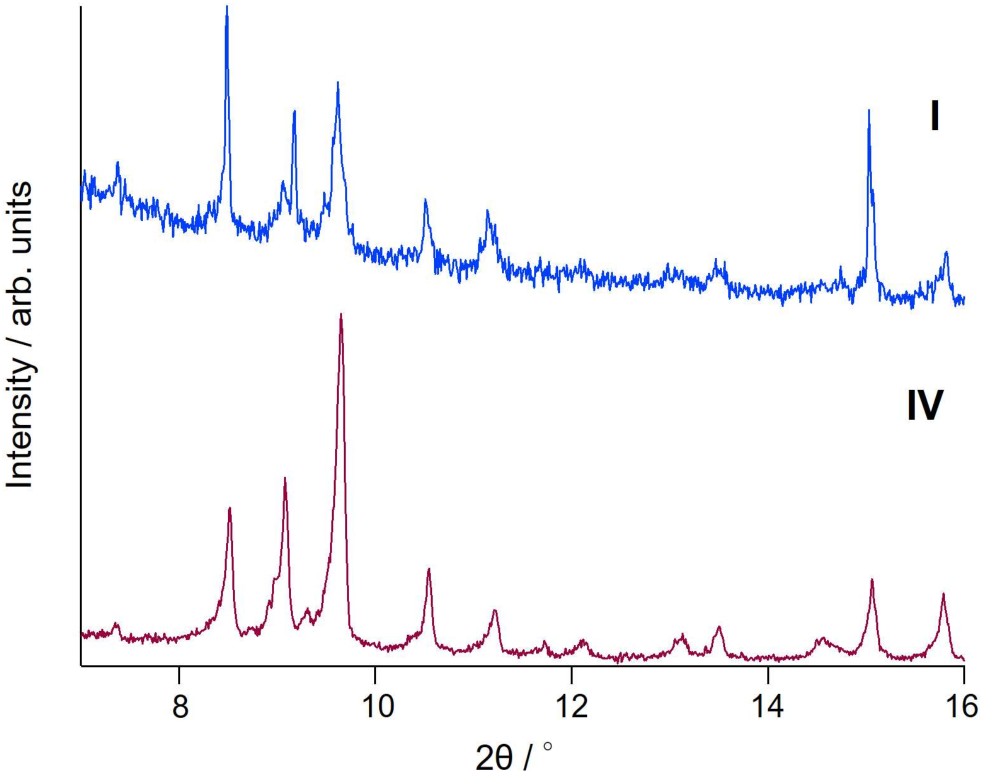

Supplementary Figure 13. Powder X-ray diffraction patterns of Tb-dinuclear (blue line) and Tb-polymer after heat treatment (dark red line).

The PXRD pattern of heat-treated Tb-polymer was matched with that of Tb-dinuclear (Supplementary Figure 13). The identification using IR and elemental analyses also confirmed the formation of Tb-dinuclear (bellow). The molar ratio between 4,4'-bpy and $Tb(tmh)_3$ core unit in Tb-polymer is calculated to be 1:1, on the other hand, the molar ratio in Tb-dinuclear is 1:2. After the depolymerization, the elimination of 4,4'-bpy leftover part is required. Crystal weight loss correspondent to the elimination of 4,4'-bpy was observed in TG measurements (Supplementary Figure 12). This partial reversible polymerization can be repeated, and the cycle is shown in Supplementary Figure 14. We also observed the PXRD signal of [Tb_2tmh_6] as stoichiometric byproduct after one cycle transformation (Supplementary Figure 15).

[$Tb_2(tmh)_6$(4,4'-bpy)] (IV: Tb-polymer after heat treatment): IR (ATR) $\tilde{\upsilon}$ = 2836–3002 (m, C–H), 1591 (m, C–N), 1572 cm^{-1} (s, C=O); elemental analysis (%): calcd. for $C_{76}H_{122}N_2O_{12}Tb_2$: C, 58.01; H, 7.81; N, 1.78; found: C, 57.64; H, 7.80; N, 1.80.

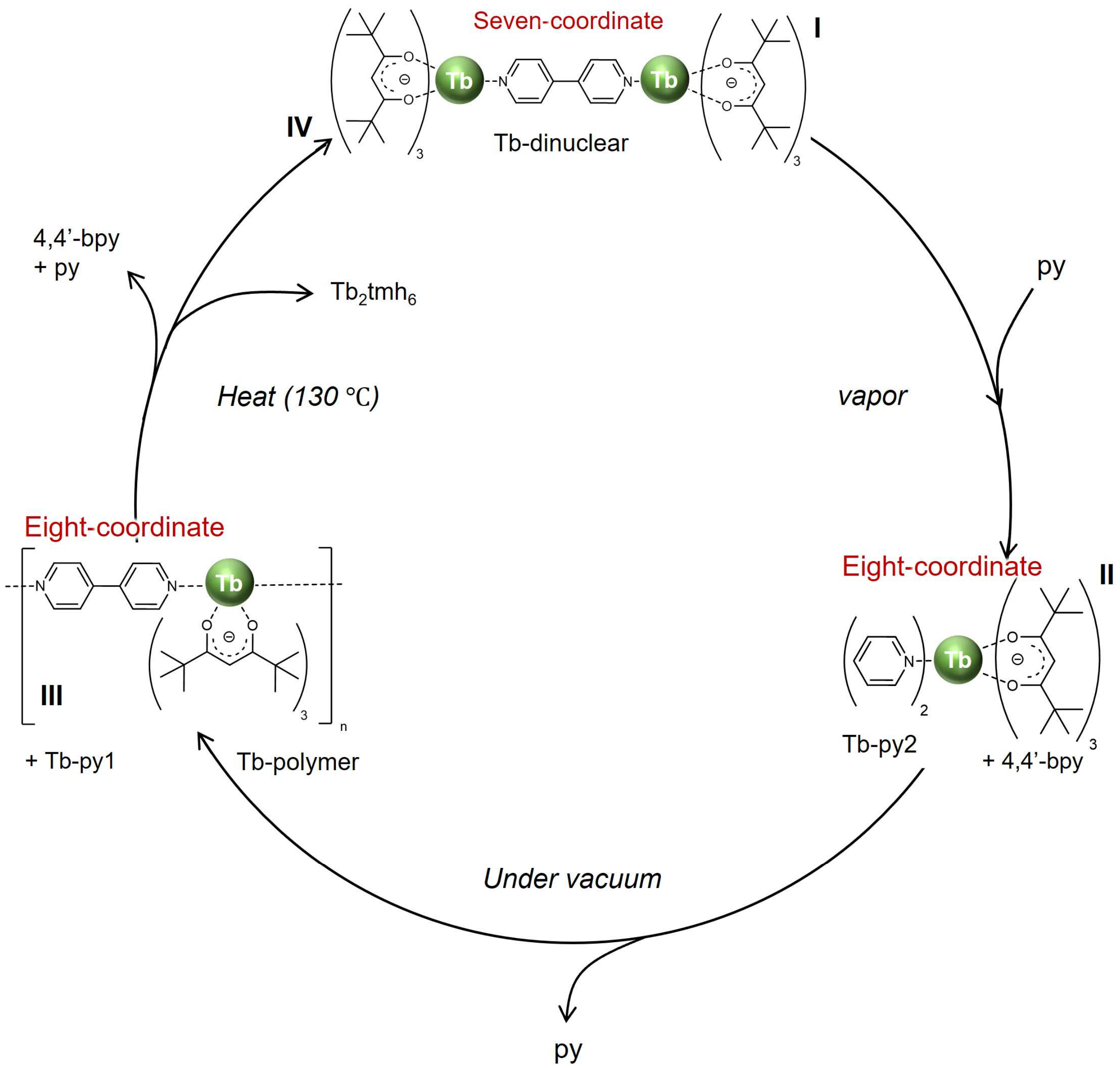

Supplementary Figure 14. Illustration of partial reversible polymerization cycle. py, pyridine; 4,4'-bpy: 4,4'-bipyridine.

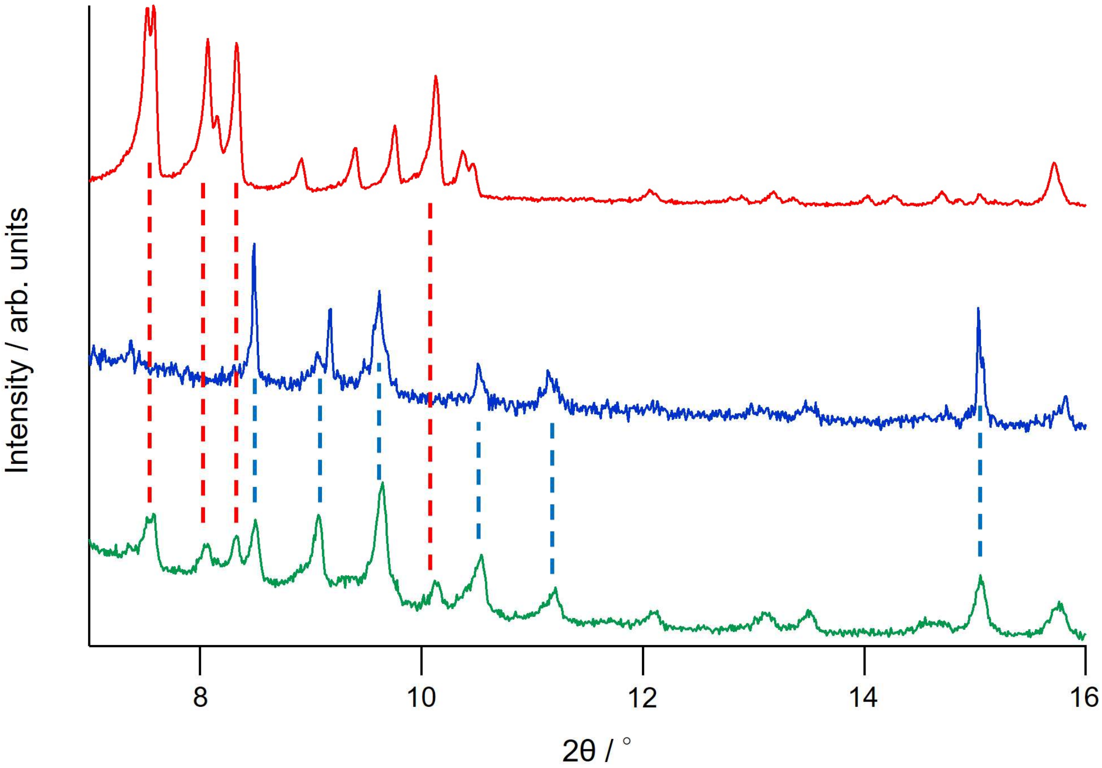

Supplementary Figure 15. Powder X-ray diffraction patterns of transformed Tb-dinuclear after one cycle (IV: green line), Tb-dinuclear (I: initial state, blue line) and precursor [$Tb_2(tmh)_6$] (red line). Dashed lines included for easier identification of matched structures. tmh, 2,2,6,6-tetramethyl-3,5-heptanedionato.

After one transformation cycle (IV: Tb-dinuclear) is completed, [Tb_2tmh_6] is formed as stoichiometric byproduct from the heating process of [$Tb(tmh)_3(py)_1$][6].

Supplementary Note 7: Energy migration experiments (control)

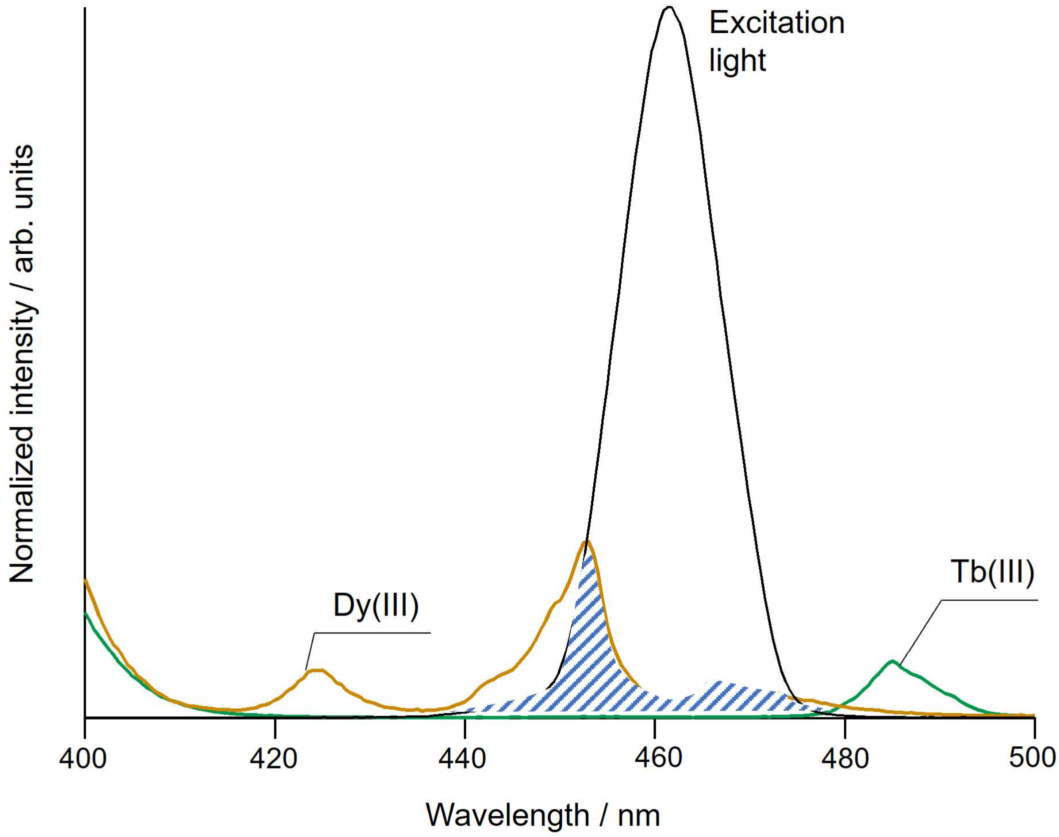

Supplementary Figure 16. Excitation spectra of Dy-polymer (dark yellow line) and Tb-polymer (dark green line) in comparison to the profile of the excitation light (Halogen lamp with 460 nm band pass filter: λ_{ex} = 460 ± 10 nm).

The excitation light at 460 ± 10 nm is mainly capable of exciting Dy(III) crystals through the direct 4*f*-4*f* absorption of Dy(III) ion. Tb(III) crystals are hardly excited using this light source.

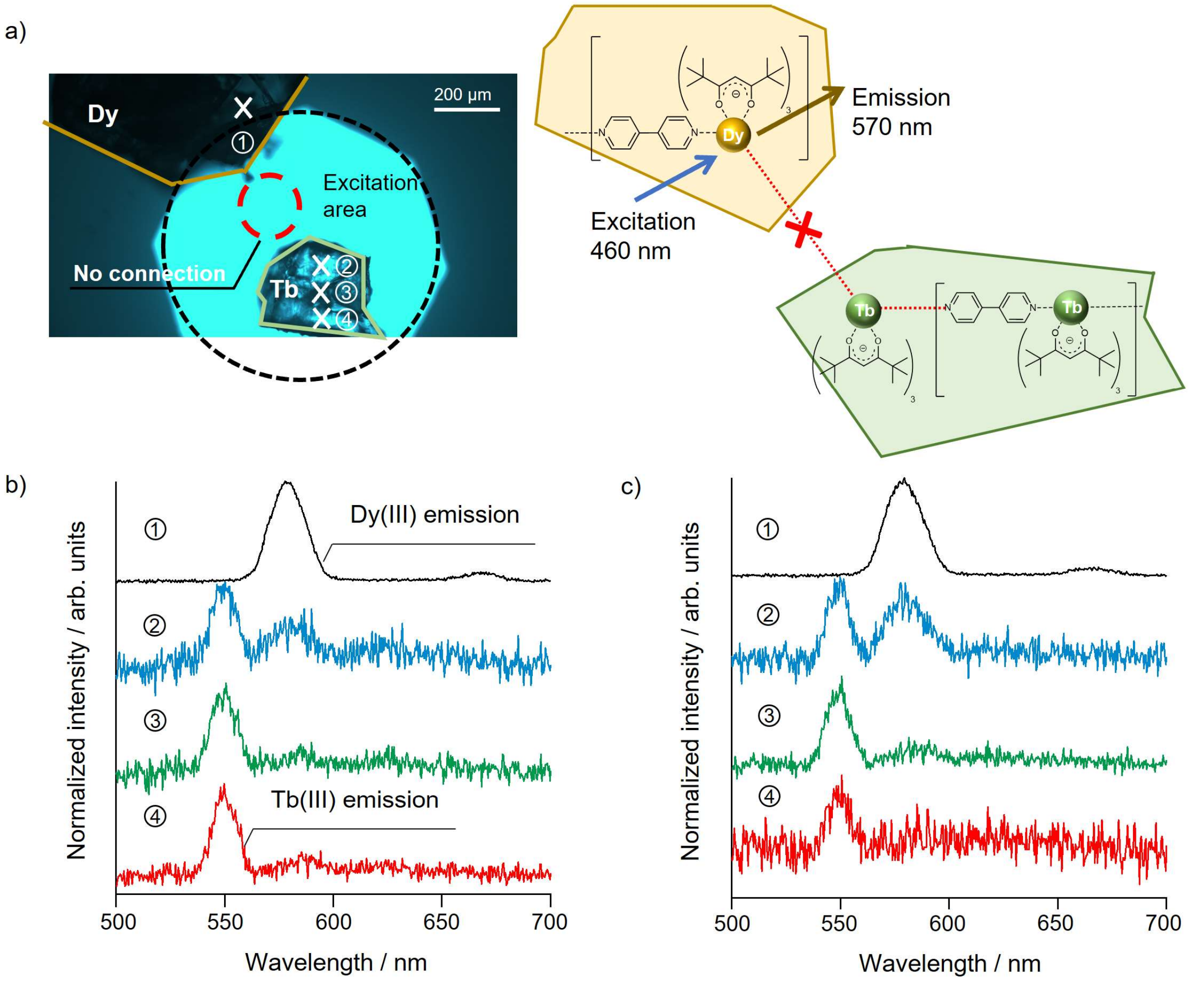

Supplementary Figure 17. a) Photophysical measurement schematic diagram for control experiment. The crystal sample is excited using a 460 nm light source for direct Dy(III) excitation. Emission spectra were measured by spatially resolved microspectroscopy and normalized at their peak top. Position-depended emission spectra in position 1 (black line), position 2 (blue line), position 3 (green line) and position 4 (red line) of the b) dinuclear system (before transformation) and c) coordination polymer (after transformation).

For the control experiment, we positioned the Tb(III) crystal and Dy(IIII) crystal separated by 100 μm to check the possibility of contamination from one crystal to the other. The emission spectra measured at different positions before and after transformation are very similar, and with very weak emission bands observed from position 2, 3, and 4. This indicates that the Tb(III)-based emission is correspondent to the weak direct excitation of Tb(III) ion at 460 nm, not from energy migration from Dy(III) crystal (check below).

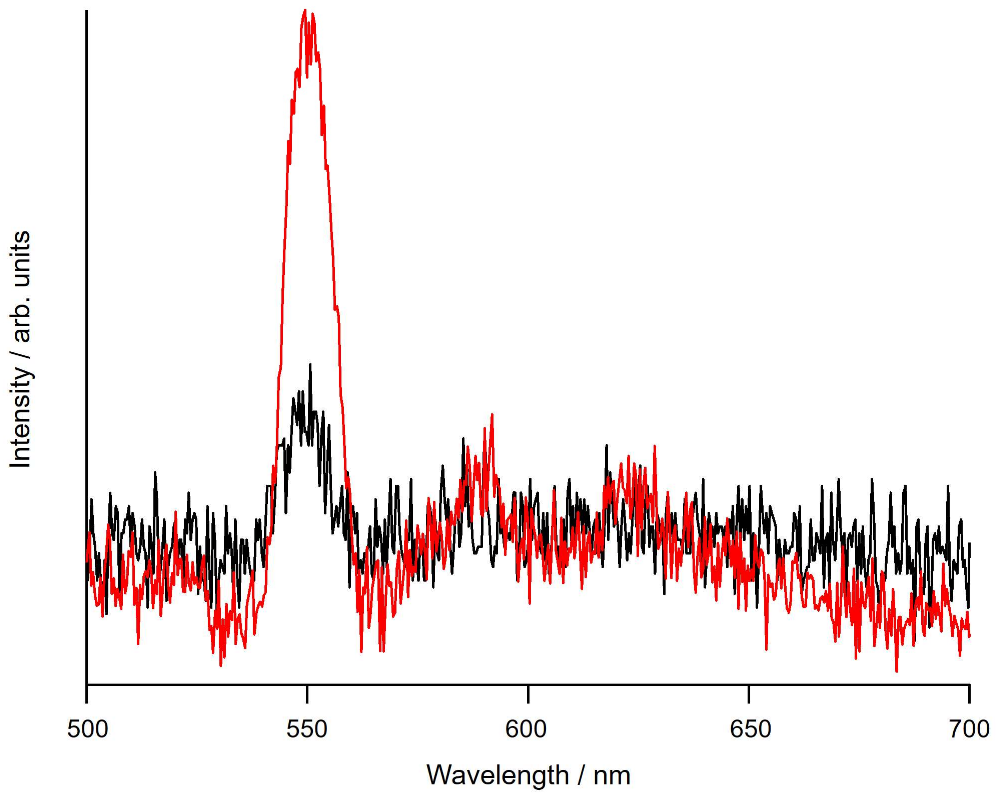

Supplementary Figure 18. Emission spectra of Tb-polymer (after transformation) excited using a 460 nm light source of control experiment (black line) and connected Dy(III)-Tb(III) crystals (red line) at each crystal system position 4. Not normalized.

In the control experiment, large separation between Dy(III) and Tb(III) crystals results in weak direct Tb(III) excitation at 460 nm (black line). Conversely, for the connected crystal, stronger emission derived from Tb(III) ion is observed. We consider that the energy migration is the dominated process for Tb(III) ion excitation.

Supplementary Note 8: Energy migration experiments (different surface connections)

The energy migration of the Tb(III)-Dy(III) connected crystal was measured using different surface connections to investigate the dependence in the energy migration efficiency. Point connected crystals at (-1 2 1) face showed stronger Tb(III) emission than point connected crystals at (1 1 0) face in two positions (Supplementary Figure 19c-d). Here, (-1 2 1) face follows the same direction as 4,4'-bpy (Supplementary Figure 19e). On the other hand, (1 1 0) face is almost perpendicular to the direction of 4,4'-bpy in the crystal (Supplementary Figure 19f). The structural transformation depends on the direction of 4,4'-bpy since it is the coordinated ligand connecting Ln(III) ions. We propose that structural transformation following the 4,4'-bpy ligand in the crystal connection enhances the linkage between the crystals and facilitates the energy transfer process.

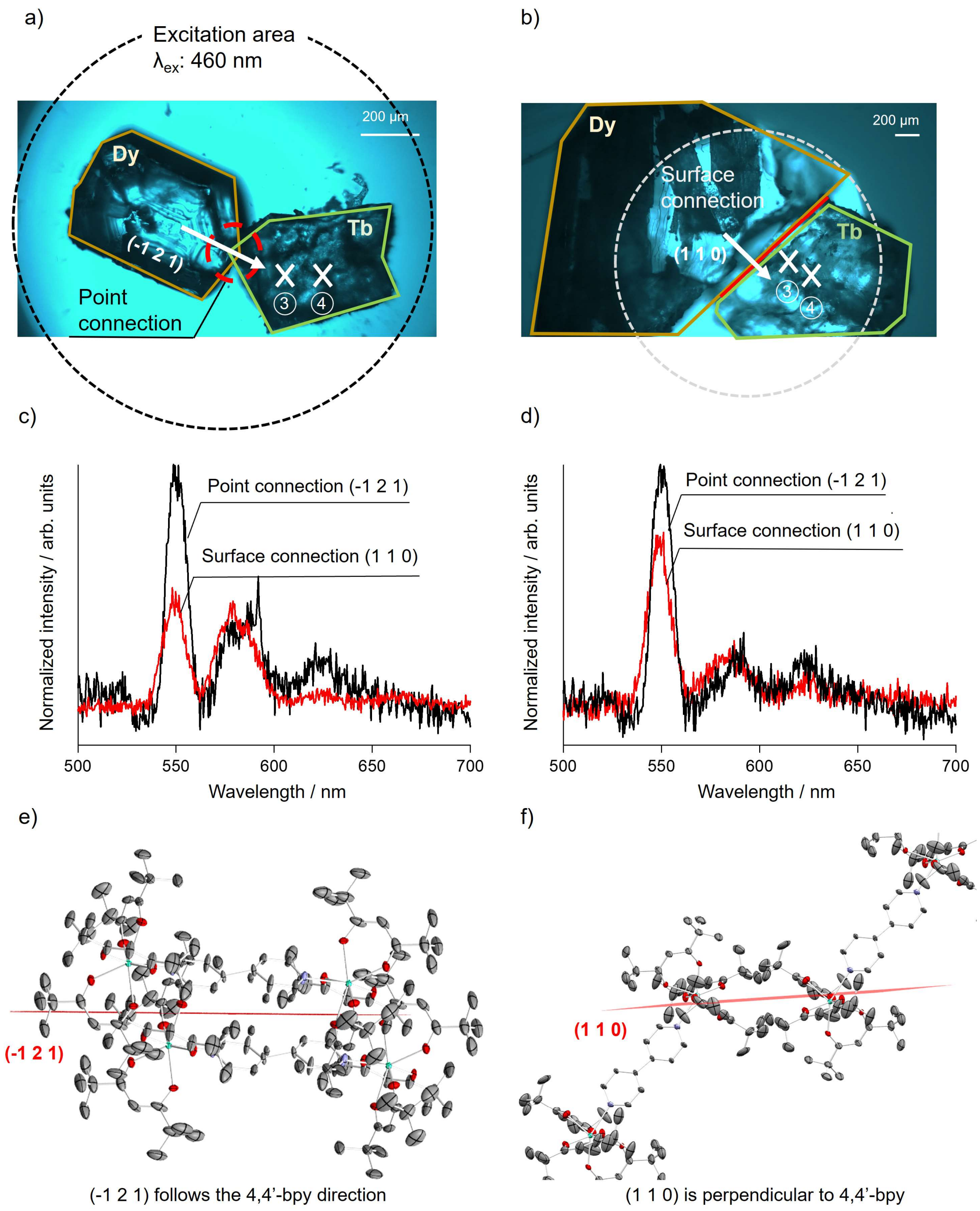

Supplementary Figure 19. a) Photophysical measurement schematic diagram of point connected crystals. b) Photophysical measurement schematic diagram of surface connected crystals. The crystal samples are excited using a 460 nm light source for direct Dy(III) excitation. Emission spectra were measured by spatially resolved microspectroscopy at the cross indication. Connected faces are represented by arrows with their correspondent labels. Emission spectra of transformed crystals normalized at 570 nm (Dy(III) based emission) in c) position 3 (closer to Dy(III) crystal) and d) position 4 (middle of Tb(III) crystal). e) Perspective views of [$Tb_2(tmh)_6$(4,4'-bpy)] dinuclear complex showing (-1 2 1) plane in red. f) Perspective views of [$Tb_2(tmh)_6$(4,4'-bpy)] dinuclear complex showing (1 1 0) plane in red.

Supplementary Note 9: Energy migration experiments (laser excitation)

The energy migration of the Tb(III)-Dy(III) connected crystal was measured using a spatially resolved microspectroscopic system for emission detection at specific regions of the merged crystal. The spatial resolution is estimated to be 20 µm because we employed the objective lens 5× and a pinhole with a diameter of 100 µm on the focal image position before the spectrometer. However, an expanded light detection is observed up to a radius of 100 µm on the focal image position, probably due to crystalline optical disorder. Here, the second harmonic generation (SHG; 452 ± 2 nm) of a mode-locked Ti:sapphire laser (904 nm, 80 MHz, 100 fs) was employed as an excitation source (Supplementary Figures 20-21). The exposure time of the spectrometer was set to 10 s and the excitation light was cut by a long-pass edge filter at 480 nm. The excitation and detection sources were separated by at least 10 µm in order to investigate the energy migration between crystals (Supplementary Figure 22).

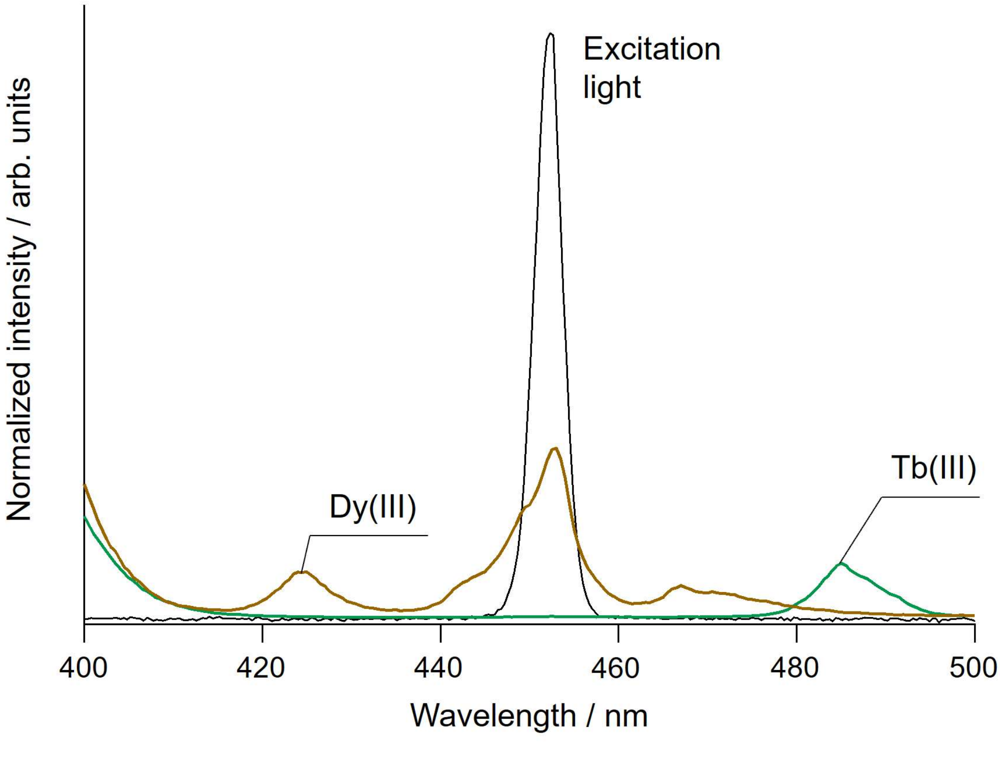

Supplementary Figure 20. Excitation spectra of Dy-polymer (dark yellow line) detected at 571 nm and Tb-polymer (dark green line) detected at 541 nm in comparison to the profile of the excitation light (SHG of the mode-locked Ti:sapphire laser: λ_{ex} = 452 ± 2 nm).

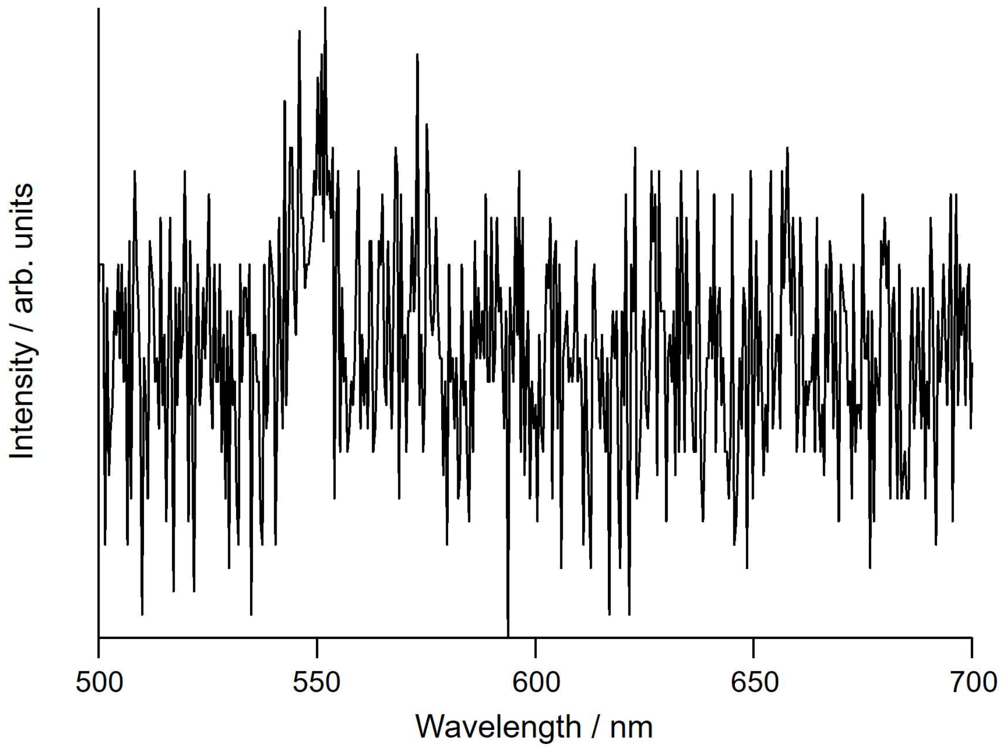

Supplementary Figure 21. Emission of Tb-dinuclear excited at 452 nm (SHG of the mode-locked Ti:sapphire laser: λ_{ex} = 452 ± 2 nm).

Supplementary Figures 20 and 21 confirm the absence of absorption and emission of Tb(III) crystals under the laser excitation (SHG of the mode-locked Ti:sapphire laser: λ_{ex} = 452 ± 2 nm).

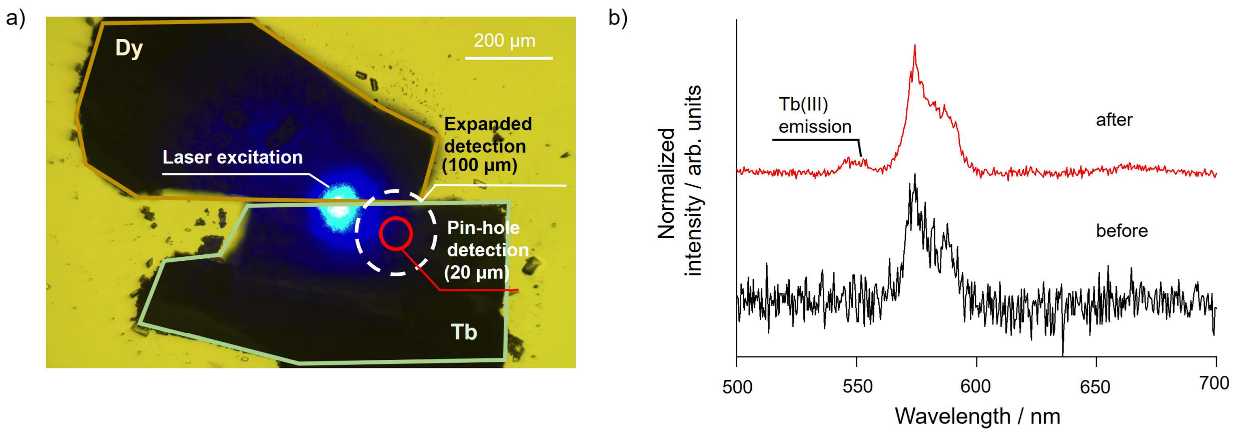

Supplementary Figure 22. a) Photophysical measurement schematic diagram for laser excited experiment. The crystal sample is excited using a 452 ± 2 nm laser light source for direct Dy(III) excitation. Emission spectra were measured by spatially resolved microspectroscopy. b) Emission spectra of the dinuclear system (before transformation: black line) and coordination polymer (after transformation: red line) at the specified position.

The unmerged Tb(III) crystal-Dy(III) crystal (before transformation) showed only Dy(III)-based emission at 570 nm (Supplementary Figure 22b). For the merged Tb(III) crystal-Dy(III) crystal, we observe Tb(III) emissions along with Dy(III) emissions (Supplementary Figure 22b), derived from the large radius of the spatially resolved microspectroscopy. The observed Tb(III) emission may only be explained by energy migration from the Dy(III) to Tb(III) crystals because only the Dy(III) ions are excited at 452 ± 2 nm using a laser system. This result is in line with results obtained in the main manuscript.

Supplementary Note 10: Energy migration experiments (mixed powder)

The energy migration was also investigated in powder conditions. Here, the Dy(III) and Tb(III) crystals are grinded into powder and mixed, and further transformed using pyridine vaporization. The energy migration was evaluated using excitation (Supplementary Figure 24) and emission spectra (Supplementary Figure 23) recorded on a HORIBA Fluorolog-3 spectrofluorometer and corrected for the response of the detector system. A xenon light diffracted using a diffraction grating with 0.5 nm slit to the wavelength of 452 ± 2 nm coupled with a 450 nm long-pass filter was employed as an excitation source for the emission spectra. Emission detection was set at 541 nm with 0.6 nm slit coupled with a 540 ± 5 nm bandpass filter for the excitation spectra.

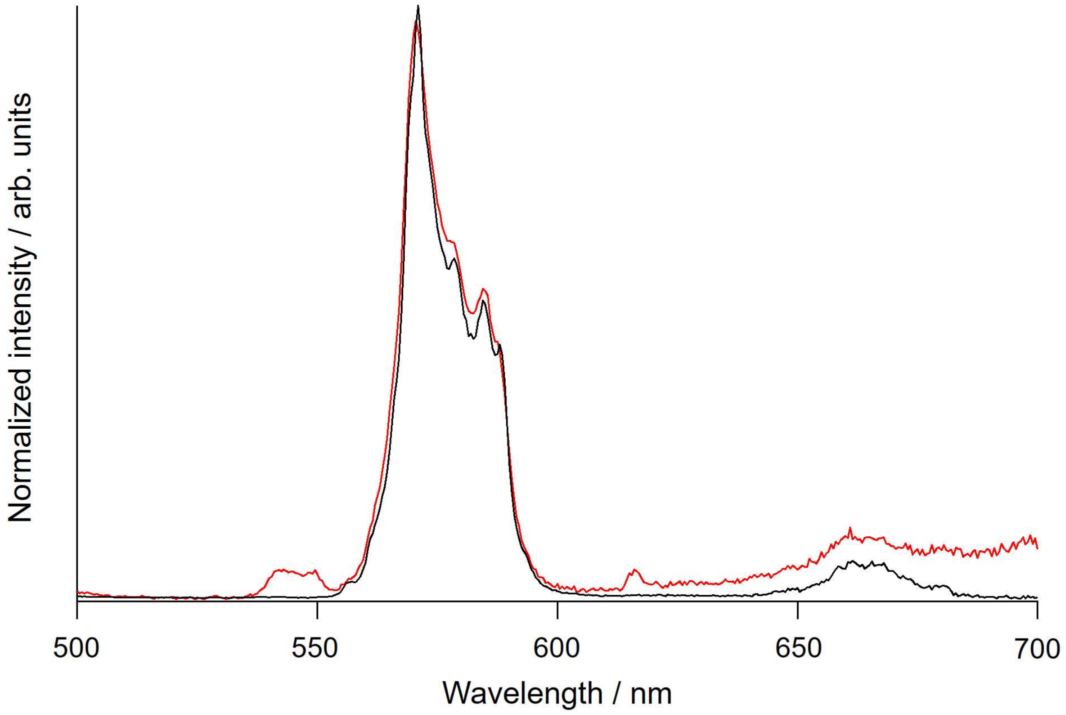

Supplementary Figure 23. Emission spectra of the Dy-polymer powder (after transformation from Dy-dinuclear: black line) and Tb-polymer/Dy-polymer mixed powder (after transformation from Tb-dinuclear and Dy-dinuclear mixed powders: red line) excited at 452 ± 2 nm. The spectra were normalized at their peak top.

The Tb(III)-based emission at around 545 nm was observed at 452 ± 2 nm (Dy(III) direct excitation) excitation along with the Dy(III)-based emission in the mixed system. The Tb(III)-based emission cannot be observed in the 100% Dy-polymer powder. This experiment

indicates that energy migration between Dy(III) and Tb(III) powder occurs effectively. In order to further confirm the energy migration, we measured the excitation spectra based on the Tb(III) emission (545 nm).

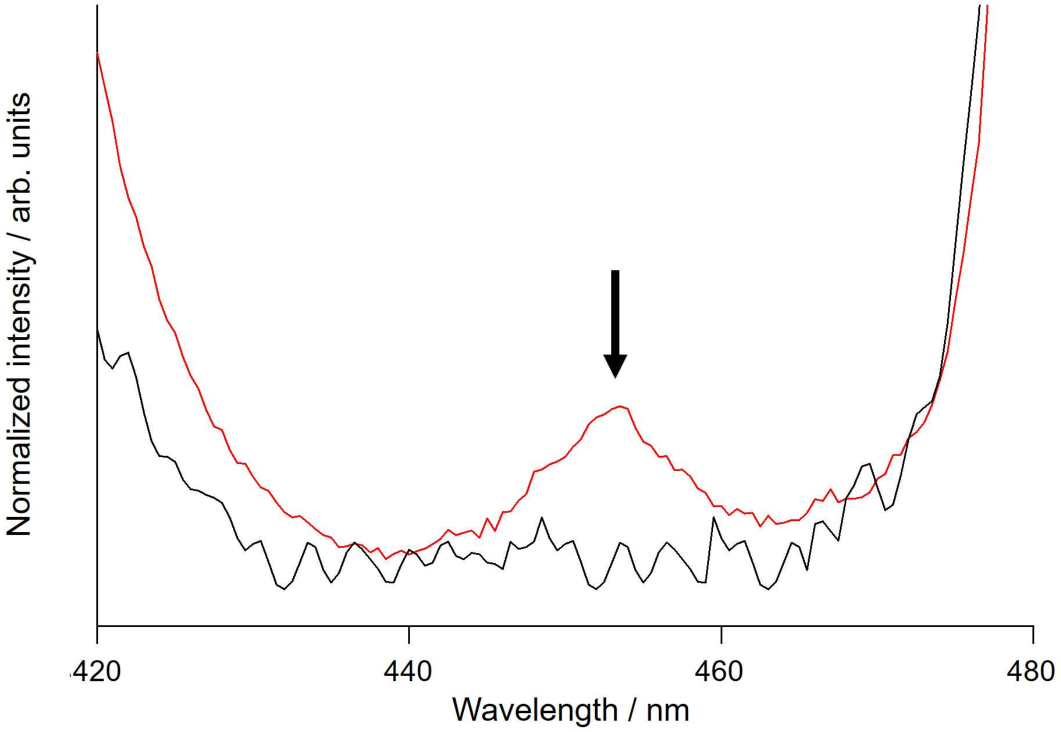

Supplementary Figure 24. Excitation spectra of the Tb-polymer powder (after transformation from Tb-dinuclear: black line) and Tb-polymer/Dy-polymer mixed powder (after transformation from Tb-dinuclear and Dy-dinuclear mixed powders: red line) detected at 541 ± 2 nm. The spectra were normalized at 484 nm (Tb(III) direct excitation: $^5D_4 \leftarrow {}^7F_6$; peak not shown).

A novel excitation band at around 453 nm is observed in the mixed system compared to the sample with 100% Tb-polymer powder. This band is assigned to be $^4I_{15/2} \leftarrow {}^6H_{15/2}$ of Dy(III) ion. This excitation band strongly supports that Tb(III) emission was enabled by Dy(III) powder energy migration.

Supplementary Note 11: Photophysical measurements

Emission and excitation spectra were recorded on a HORIBA Fluorolog-3 spectrofluorometer and corrected for the response of the detector system. Emission lifetimes (τ_{obs}) were measured using the third harmonics (355 nm) of a Q-switched Nd:YAG laser (Spectra Physics, INDI-50, fwhm = 5 ns, λ = 1064 nm) and a photomultiplier (Hamamatsu Photonics, R5108, response time ≤ 1.1 ns). The Nd:YAG laser response was monitored with a digital oscilloscope (Sony Tektronix, TDS3052, f = 500 MHz) synchronized to the single-pulse excitation. Emission lifetimes were determined from the slope of logarithmic plots of the decay profiles. The emission quantum yields of Tb(III) complexes excited at 360 ($\Phi_{\pi\text{-}\pi*}$) and 484 nm (Φ_{ff}); and Dy(III) complexes excited at 360 ($\Phi_{\pi\text{-}\pi*}$) and 455 nm (Φ_{ff}) were estimated using a JASCO F-6300-H spectrometer attached with a JASCO ILF-533 integrating sphere unit (ϕ = 100 nm) under air. The wavelength dependence of the detector response and the beam intensity of the Xe light source for each spectrum were calibrated using a standard light source. Emission lifetimes in the range of 100-390 K were measured using a cryostat (Thermal Block Company, SA-SB1905HA) and a temperature controller (Scientific Instruments, Model 9700).

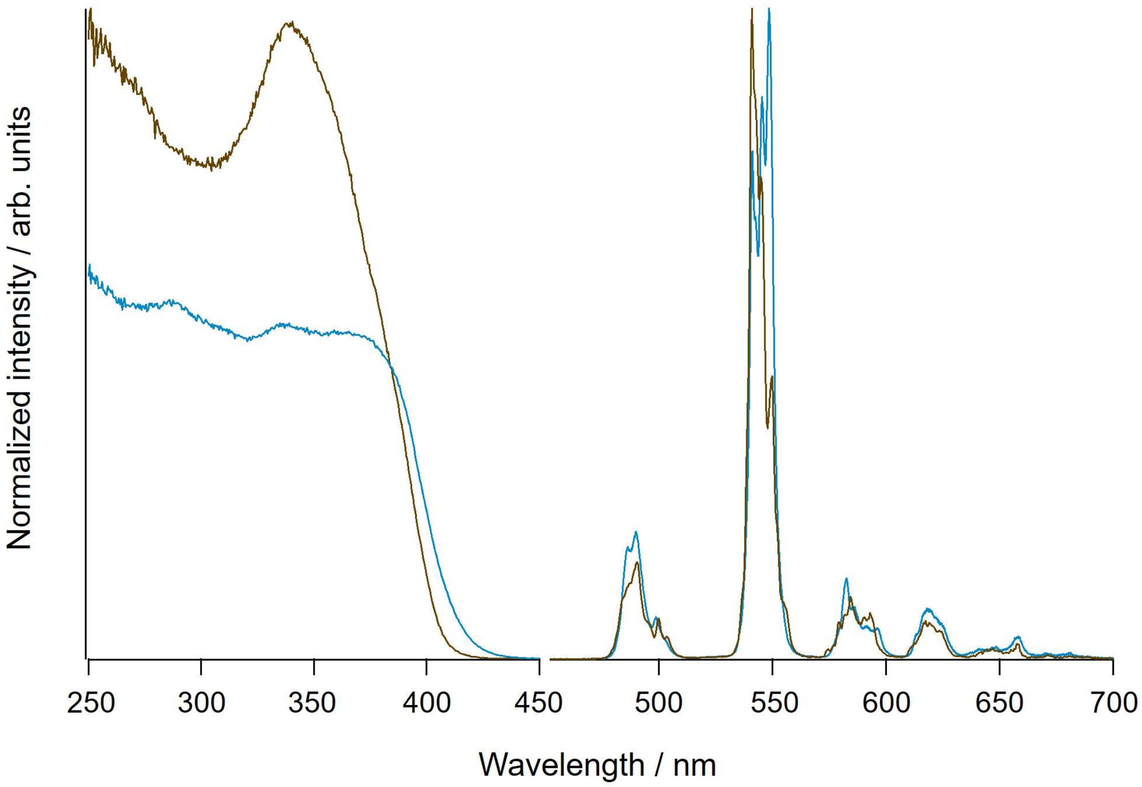

Supplementary Figure 25. Excitation (left) and emission (right) spectra of Tb-dinuclear (blue line) and Tb-polymer (brown line) in solid state at room temperature. Excitation spectra were recorded with emission at 545 nm ($^5D_4 \rightarrow {^7F_5}$) and normalized at 484 nm (Tb(III) direct excitation: $^5D_4 \leftarrow {^7F_6}$; peak not shown). Emission spectra were excited at 350 nm and normalized at their peak tops.

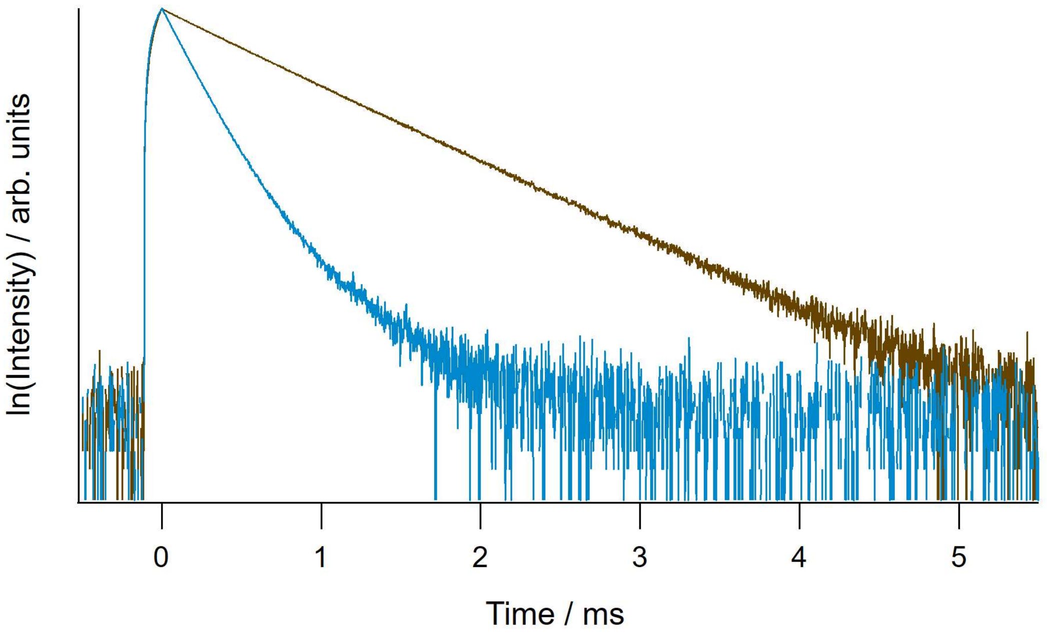

Supplementary Figure 26. Emission decay profiles of Tb-dinuclear (blue line) and Tb-polymer (brown line) under 356 nm excitation at room temperature. Detection wavelength was set to 545 nm ($^5D_4 \rightarrow {}^7F_5$). The emission lifetimes were obtained from single-exponential analyses.

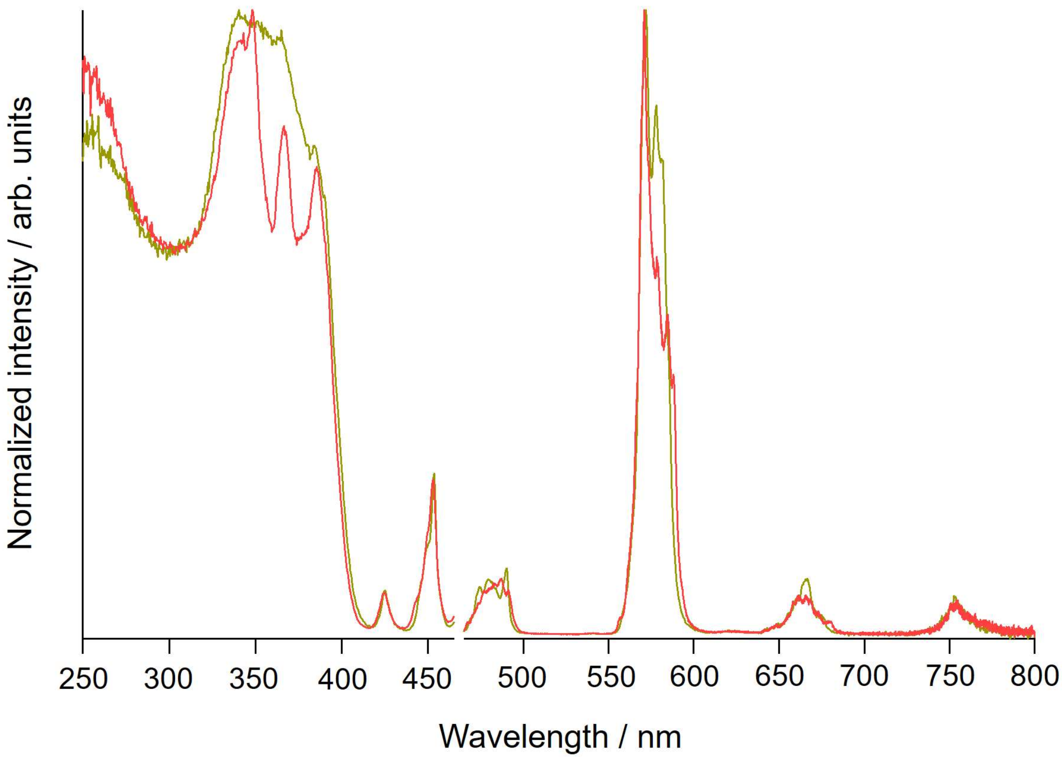

Supplementary Figure 27. Excitation (left) and emission (right) spectra of Dy-dinuclear (dark yellow line) and Dy-polymer (red line) in solid state at room temperature. Excitation spectra were recorded with emission at 571 nm ($^4F_{9/2} \rightarrow {}^6H_{13/2}$). Emission spectra were excited at 350 nm. They were normalized at their peak top.

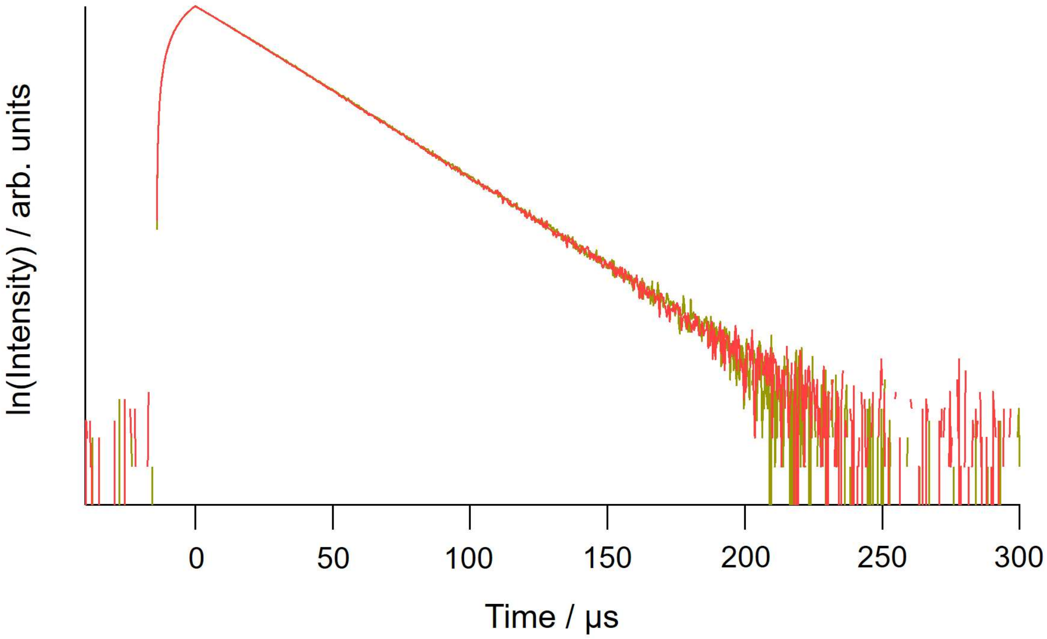

Supplementary Figure 28. Emission decay profiles of Dy-dinuclear (blue line) and Dy-polymer (brown line) under 356 nm excitation at room temperature. Detection wavelength was set to 571 nm ($^4F_{9/2}\rightarrow$ $^6H_{13/2}$). The emission lifetimes were obtained from single-exponential analyses.

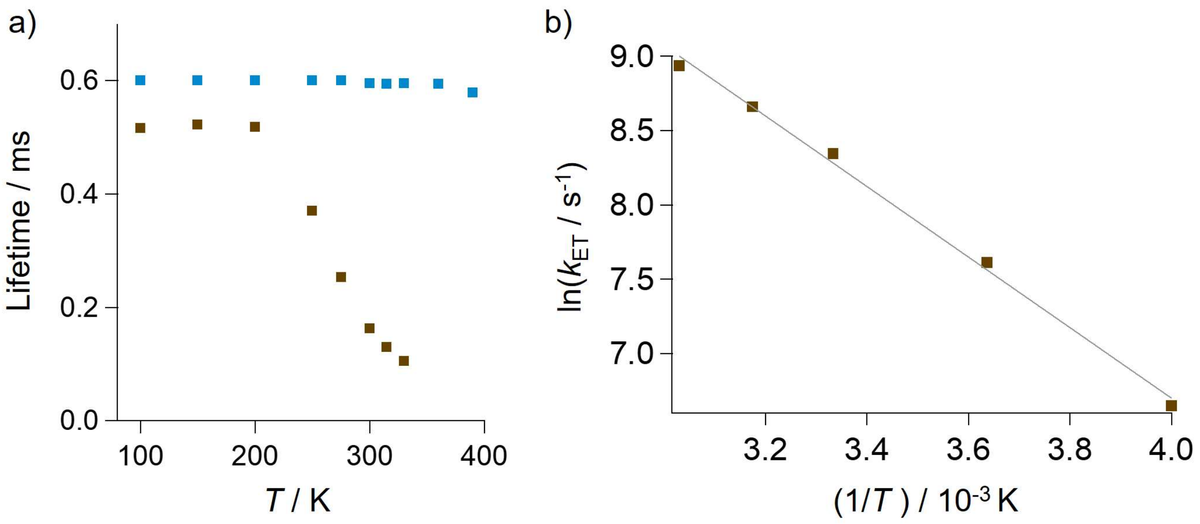

Supplementary Figure 29. a) Emission lifetime temperature-dependent derived from $^5D_4 \rightarrow ^7F_5$ for Tb-dinuclear (brown dot) and Tb-polymer (blue dot). Excitation wavelength was at 355 nm (Nd:YAG laser, 3ω). Measured in vacuum. The emission lifetimes were obtained from single-exponential analyses. b) Arrhenius analyses based on Supplementary Equation (2) of emission lifetime temperature dependence for Tb-dinuclear (dark yellow dot and yellow fitting line). Tb-polymer did not show any temperature-dependence up to 390 K.

Arrhenius equation: $\ln (1/\tau_{obs} - 1/\tau_{const}) = \ln (k_{ET}) = \ln (A) - (\Delta E/k_B T)$ (2)

where, τ_{obs} is the observed emission lifetime at each temperature; τ_{const} is the emission lifetime in which up to that temperature the lifetime obtained for such transition is constant; k_B is the Boltzmann constant.

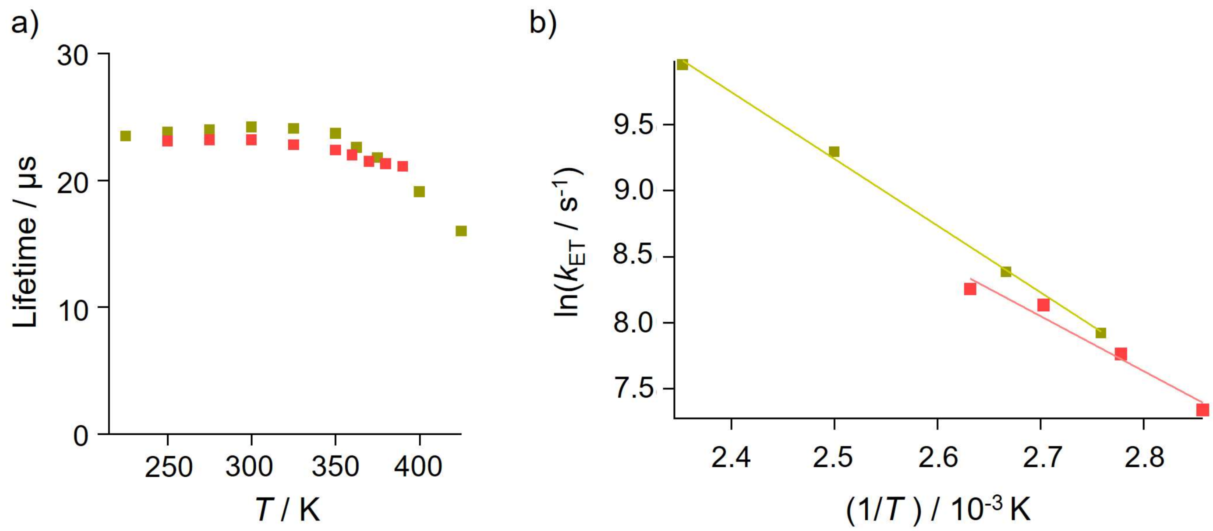

Supplementary Figure 30. a) Emission lifetime temperature-dependent derived from $^4F_{9/2}{\rightarrow}^6H_{13/2}$ for Dy-dinuclear (dark yellow dot) and Dy-polymer (red dot). Excitation wavelength was at 355 nm (Nd:YAG laser, 3ω). Measured in vacuum. The emission lifetimes were obtained from single-exponential analyses. b) Arrhenius analyses based on Supplementary Equation (2) of emission lifetime temperature dependence for Dy-dinuclear (dark yellow dot and yellow fitting line) and Dy-polymer (red dot and pink fitting line).

Supplementary Note 12: Excited states calculations

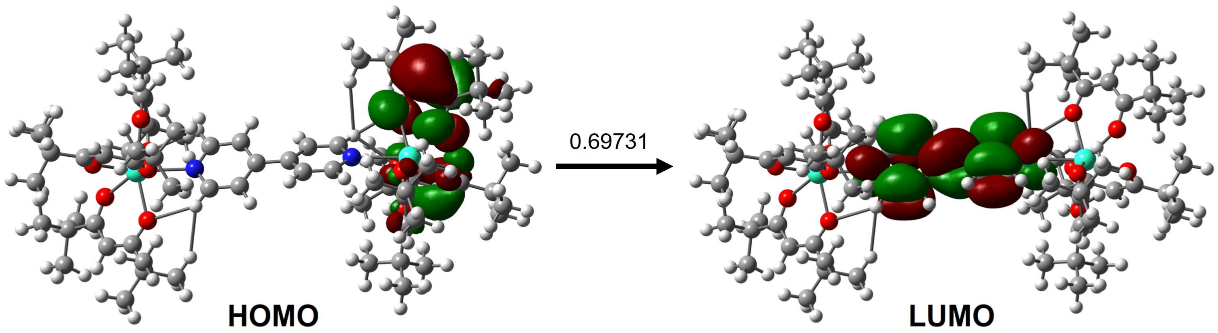

Supplementary Figure 31. Molecular orbital representations of the S_0-T_1 transition of Tb-dinuclear.

The S_0-T_1 transition for the dinuclear structure shows strong LLCT (Ligand-to-ligand charge transfer) character, with electron density transition from tmh to 4,4'-bpy ligands.

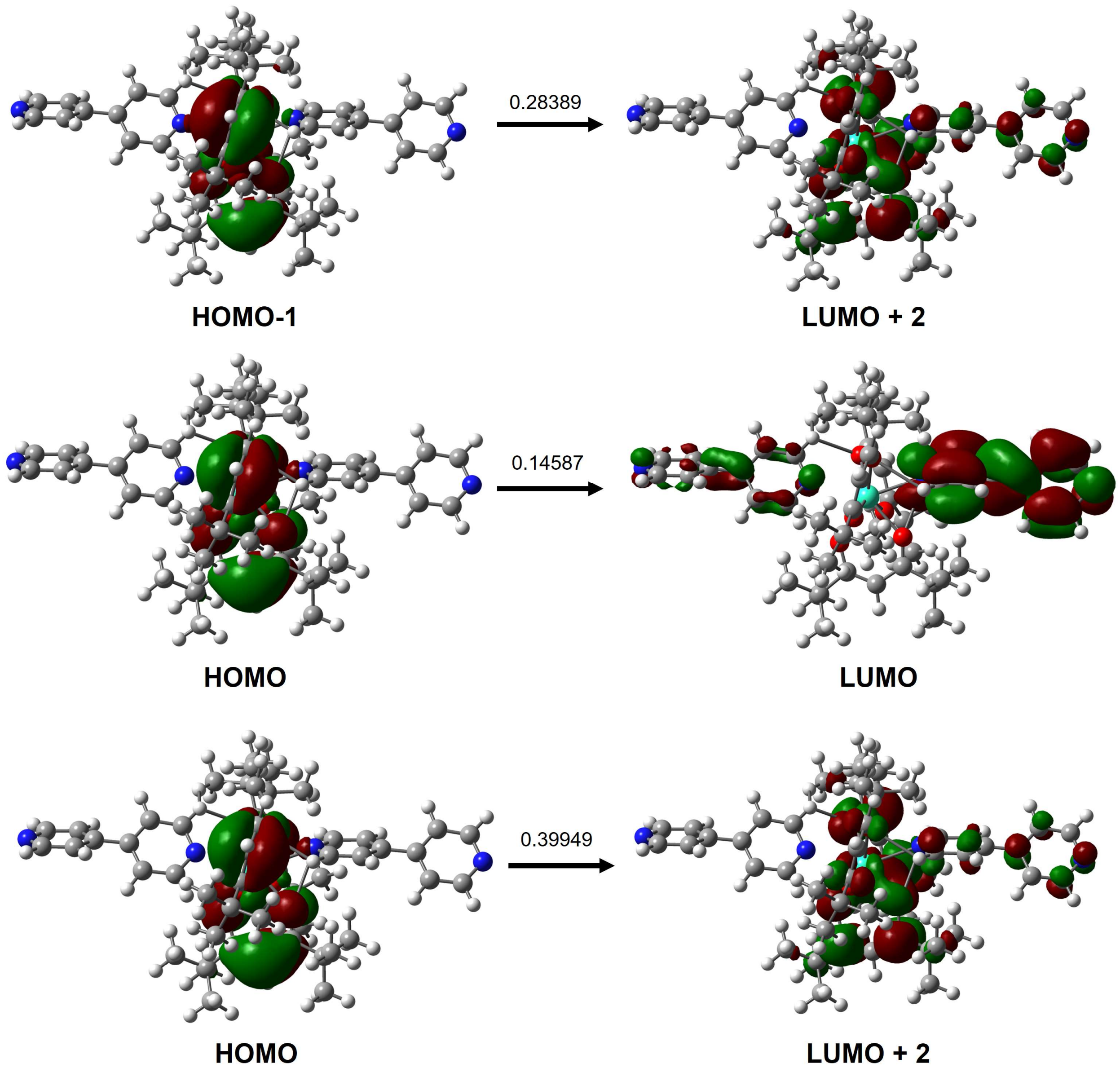

Supplementary Figure 32. Molecular orbital representations of the S_0-T_1 transition of Tb-polymer, showing the transition density probabilities.

The S_0-T_1 transition for the polymer structure shows weak LLCT (Ligand-to-ligand charge transfer) character, with electron density transition from tmh to a mix of tmh and 4,4'-bpy ligands.

Supplementary Table 7. Summary of calculated T_1 states of Tb-dinuclear and Tb-polymer.

Complex	T_1 / nm	T_1 / cm^{-1}	Character	ΔE (T_1-$^5D_4$) / cm^{-1}	ΔE (T_1-$^4F_{9/2}$) / cm^{-1}
Tb-dinuclear	431.2	23,190	Strong LLCT	2,690	2,090
Tb-polymer	412.6	24,240	Weak LLCT	3,740	3,140

Tb(III) excited state ($^5D_4$: 20,500 cm^{-1}); Dy(III) excited state ($^4F_{9/2}$: 21,100 cm^{-1}).[7]

Dipole moment in each $Tb(tmh)_3$ centre:

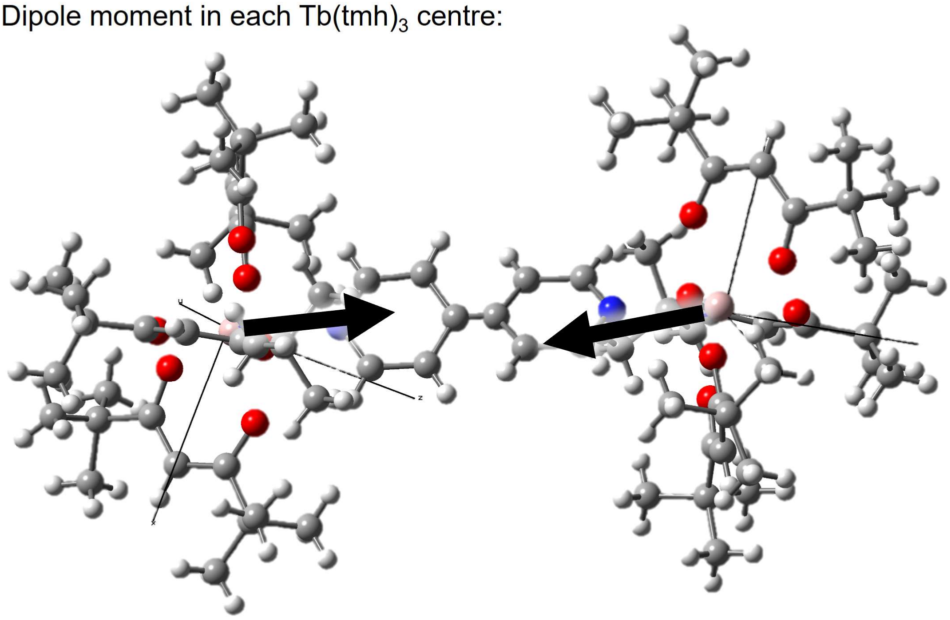

Total dipole moment in the crystal:

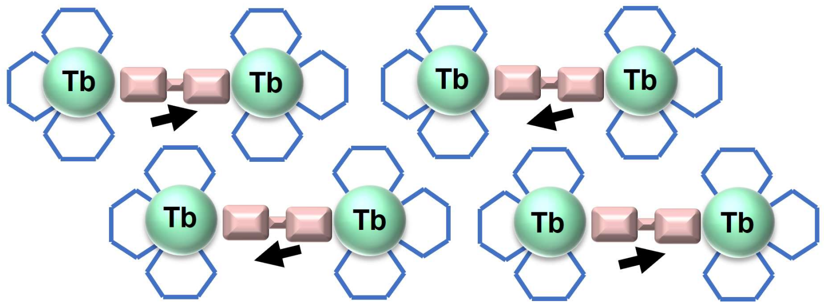

Supplementary Figure 33. Dipole moment vector calculated for each $Tb(tmh)_3$ centre based on the dinuclear structure (top). Total dipole of each dinuclear molecule in the crystal (bottom). tmh, 2,2,6,6-tetramethyl-3,5-heptanedionato.

Dipole moment in each $Tb(tmh)_3$ centre:

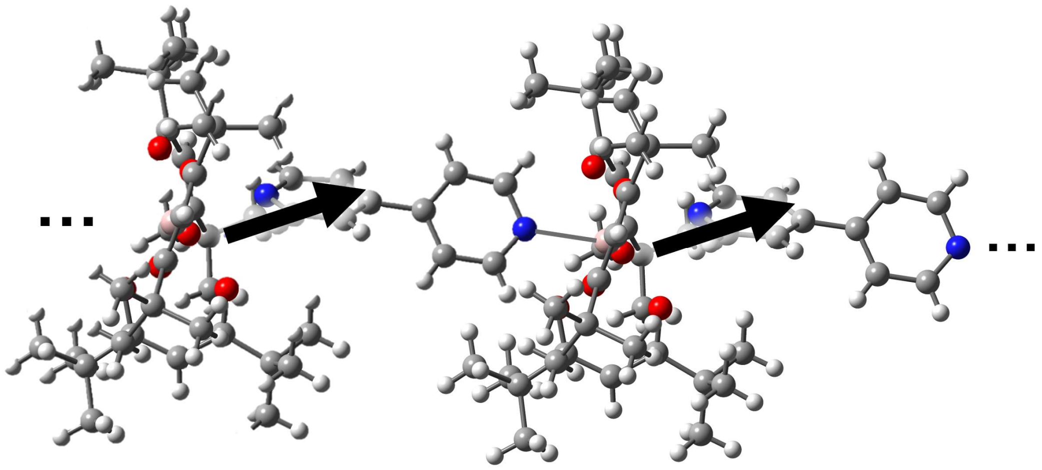

Total dipole moment in the crystal:

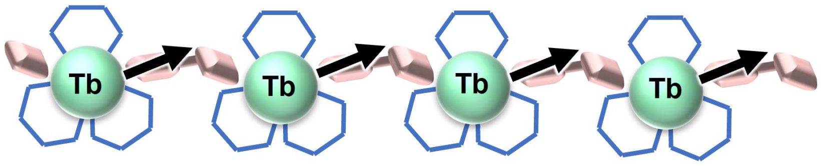

Supplementary Figure 34. Dipole moment vector calculated for each $Tb(tmh)_3$ centre based on the polymer structure (top). Total dipole of each monomer unit in the crystal (bottom). tmh, 2,2,6,6-tetramethyl-3,5-heptanedionato.

Supplementary Note 13: Ligand-based emission process in Gd-polymer

Gd(III) ions are lanthanides with excited states at higher energies (> 30,000 cm^{-1}) than the energy levels of the tmh and 4,4’-bpy ligands. Therefore, the emission for Gd(III) based complexes is derived from the singlet and triplet states of the ligands. In the case of Gd(III) complexes, the starting $GdCl_3.6H_2O$ reactant showed 99.9% purity. Due to comparable physical features, Gd(III) ions are easily contaminated with Eu(III) and Tb(III) ions. Furthermore, these coordination compounds have exceptional photosensitization efficiencies (near 100%) for Tb(III) ions, allowing them to be emitted even at 0.1% concentration. The emission derived from β-diketonate and pyridine ligands in the Gd(III) complexes is also relatively weak, which compares to the Tb(III) emission.

Here, for the Gd-polymer ($[Gd(tmh)_3(4,4'\text{-bpy})]_n$) structure a broad emission was observed, indicating aa strong delocalized charge-transfer character in the coordination polymer structure. In comparison, mononuclear $[Gd(tmh)_3(py)_2]$ with localized excited electron densities showed sharp bands with corresponding vibrational bands.

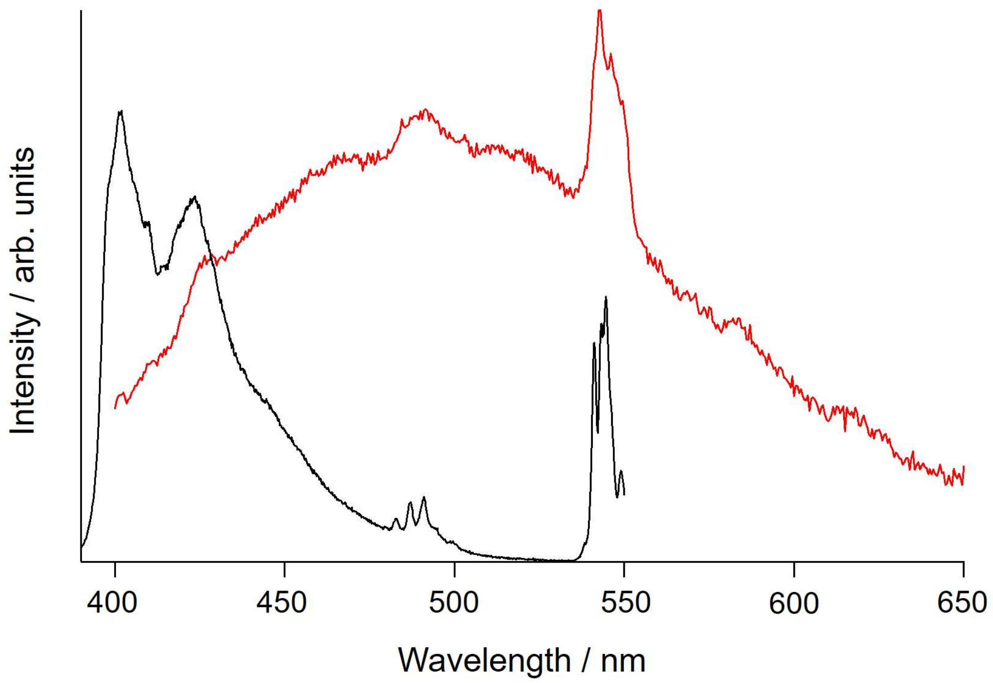

Supplementary Figure 35. Emission spectra of Gd-polymer ($[Gd(tmh)_3(4,4'\text{-bpy})]_n$: red line) and mononuclear $[Gd(tmh)_3(py)_2]$ (black line) in solid state at 300 K in vacuum. The samples were excited at 380 nm. Emission peaks at around 485 nm and 545 nm are assigned to Tb(III) ion (impurity). Mononuclear $[Gd(tmh)_3(py)_2]$ (black line) was measured at 100 K because of poor emission intensities at 300 K. tmh, 2,2,6,6-tetramethyl-3,5-heptanedionato; 4,4'-bpy, 4,4'-bipyridine; py, pyridine.

Supplementary References

1. Stabnikov, P. A., Zharkova, G. I., Smolentsev, A. I., Pervukhina, N. v. & Krisyuk, V. v. Structure and properties of terbium(III) dipivaloylmethanate and its adducts with Bipy and Phen. *Journal of Structural Chemistry* **52**, 560–567 (2011).
2. Sheldrick, G. M. A short history of SHELX. *Acta Crystallographica Section A: Foundations of Crystallography* **64**, 112–122 (2008).
3. Dolomanov, O. v., Bourhis, L. J., Gildea, R. J., Howard, J. A. K. & Puschmann, H. OLEX2: A complete structure solution, refinement and analysis program. *Journal of Applied Crystallography* **42**, 339–341 (2009).
4. a: Casanova, D., Cirera, J., Llunell, M., Alemany, P., Avnir, D. & Alvarez, S. Minimal Distortion Pathways in Polyhedral Rearrangements, *Journal of the American Chemical Society*, **126**, 1755-1763 (2004). b: Pinsky, M. & Avnir, D. Continuous Symmetry Measures. 5. The Classical Polyhedra. *Inorganic Chemistry* **37**, 5575–5582 (1998).
5. Pedro Paulo Ferreira da Rosa CCDC 1979574: Experimental Crystal Structure Determination, 2021, DOI: 10.5517/ccdc.csd.cc24fx7r.
6. Pedro Paulo Ferreira da Rosa CCDC 1979573: Experimental Crystal Structure Determination, 2021, DOI: 10.5517/ccdc.csd.cc24fx6q.
7. Carnall, W. T., Fields, P. R. & Rajnak, K. Electronic Energy Levels of the Trivalent Lanthanide Aquo Ions. III. Tb^{3+}. *The Journal of Chemical Physics* **49**, 4447–4449 (1968).
