# Supplementary material for: Preparation of photonic molecular trains via soft-crystal polymerization of lanthanide complexes
Source: Nat Commun. 2022 Jul 5;13:3660. doi: 10.1038/s41467-022-31164-z (PMC9256636; doi:10.1038/s41467-022-31164-z)
Supplement: Supplementary file 3 — Description of Additional Supplementary Files [file 41467_2022_31164_MOESM3_ESM.pdf]

## Description of Additional Supplementary files

File name: Supplementary Movie 1

Description: Connection of Tb(III) crystal and Dy (III) crystal via pyridine vaporisation.
